# Liposomal Formulations of Novel BODIPY Dimers as Promising Photosensitizers for Antibacterial and Anticancer Treatment

**DOI:** 10.3390/molecules29225304

**Published:** 2024-11-10

**Authors:** Weronika Porolnik, Magdalena Ratajczak, Aleksandra Mackowiak, Marek Murias, Malgorzata Kucinska, Jaroslaw Piskorz

**Affiliations:** 1 Chair and Department of Inorganic and Analytical Chemistry, Poznan University of Medical Sciences, Rokietnicka 3, 60-806 Poznan, Poland; 2 Chair and Department of Toxicology, Poznan University of Medical Sciences, Rokietnicka 3, 60-806 Poznan, Poland; marek.murias@ump.edu.pl (M.M.); kucinska@ump.edu.pl (M.K.); 3 Doctoral School, Poznan University of Medical Sciences, Bukowska 70, 60-812 Poznan, Poland; 4 Chair and Department of Genetics and Pharmaceutical Microbiology, Poznan University of Medical Sciences, Rokietnicka 3, 60-806 Poznan, Poland; mratajczak@ump.edu.pl

**Keywords:** anticancer research, bacterial inactivation, BODIPY, dimers, drug delivery, heavy atom effect, liposomes, photochemistry, photodynamic therapy

## Abstract

Synthesis, photochemical properties, liposomal encapsulation, and in vitro photodynamic activity studies of novel BODIPY dimer connected at *meso-meso* positions and its brominated and iodinated analogs were described. UV-Vis measurements indicated that the dimeric structure of obtained BODIPYs did not significantly influence the positions of the absorption maxima. Emission properties and singlet oxygen generation studies revealed a strong heavy atom effect of brominated and iodinated BODIPY dimers, manifested by fluorescence intensity reduction and increased singlet oxygen generation ability compared to analog without halogen atoms. For the in vitro photodynamic activity studies, dimers were incorporated into two different types of liposomes: positively charged DOTAP:POPC and negatively charged POPG:POPC. The photoinactivation studies revealed high activity of brominated and iodinated dimers incorporated into DOTAP:POPC liposomes on both Gram-positive *Staphylococcus aureus* and Gram-negative *Escherichia coli*. Anticancer studies on human breast adenocarcinoma MDA-MB-231 and human ovarian carcinoma A2780 cells revealed that DOTAP:POPC liposomes containing brominated and iodinated dimers were active even at low nanomolar concentrations. In addition, they were more active against MDA-MB-231 cells than A2780 cells, which is particularly important since the MDA-MB-231 cell line represents triple-negative breast cancer, which has limited therapeutic options.

## 1. Introduction

**Figure 1:**
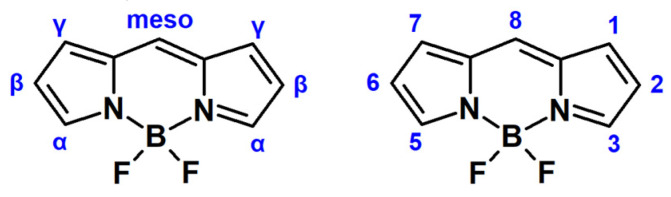
Chemical structure of BODIPY dyes with atoms positions and numbering.

Photodynamic therapy (PDT) is a medical modality mainly used in oncology, dermatology, and dentistry to treat various cancerous and non-cancerous diseases, as well as microbial infections. A light-sensitive substance called a photosensitizer is a critical element of this treatment. Upon exposure to light of an appropriate wavelength, photosensitizer generates free radicals, and reactive oxygen species (ROS), causing the death of target cells [1,2,3]. Many substances, including naturally occurring compounds such as hypericin and curcumin, and semi or fully synthetic dyes, e.g., porphyrins and phthalocyanines, have been used as photosensitizers for PDT. One of the most promising photosensitizers are derivatives of 4,4-difluoro-4-bora-3a,4a-diaza-*s*-indacene called boron-dipyrromethenes (BODIPYs) [4,5] (Figure 1).

BODIPY dimers are highly diverse dyes in which two subunits can be fused or connected at different positions (α, β, γ, or *meso*) directly and through various non-conjugated and conjugated linkers. Fluorescent BODIPY dimers possess favorable and tunable photophysical properties for bioimaging and diagnostic applications [6,7]. Dimers of BODIPY dyes have also been used to prepare self-organizing assemblies and nanoparticles for fluorescence or photoacoustic imaging [8,9]. Efficient energy transfer between BODIPY subunits in asymmetric dimers can provide energy transfer applications for photocatalysis and electronic devices such as organic solar cells [10,11]. Iodinated BODIPY dimers were studied as organic triplet sensitizers for applications in photovoltaics, photocatalysis, and various energy and charge transfer processes [11,12]. Recently, dimers with the orthogonal arrangement of two BODIPY subunits gained much attention because of the occurring spin–orbit charge-transfer intersystem crossing mechanism (SOCT-ISC). It enables high singlet oxygen generation quantum yields (Φ_Δ_) without heavy atoms in the structure. Heterodimers directly connected at *meso*-α or *meso*-β-positions revealed this effect, providing high Φ_Δ_ values but also good fluorescence intensity, providing dual functionality for PDT and fluorescence imaging [13,14,15]. Recently, orthogonal homodimers linked at γ-γ positions with efficient singlet oxygen generation have also been prepared [16,17]. The quantum yield of singlet oxygen in orthogonal BODIPY dimers can be significantly influenced by the solvent polarity with efficient intersystem crossing in moderate polarity solvents [18]. It was also found that singlet oxygen production in orthogonal *meso*-β dimers can be easily modified by a push-pull effect resulting from the presence of strong electron-withdrawing or electron-donating groups at the *meso* position of one BODIPY unit. Different singlet oxygen generation values in chloroform from 0.11 to 0.88 were obtained depending on the substituents. Also, the styryl substituent at the 3 or 5 positions caused the asymmetrical extension of π-conjugation, providing moderate yields of both singlet oxygen generation and fluorescence for potential dual functionality of photodynamic therapy and imaging [14]. The formation of BODIPY dimers in a specific position also allows a balance between fluorescence and singlet oxygen generation to obtain dyes with properties beneficial for phototheragnosis [13]. On the other hand, orthogonal BODIPY dimers usually absorb light of relatively short wavelengths and act through the oxygen-dependent type II photodynamic mechanism. Thus, Guo et al. synthesized other *meso* -α-linked heterodimers, which showed long-wavelength absorption and the ability to form both superoxide anion (O_2_^−•^) and singlet oxygen (^1^O_2_). The production of free radicals in the type I photodynamic therapy mechanism is essential for reducing the oxygen dependence of type II photosensitizers [19]. Noteworthy, Teng et al. prepared non-orthogonal α-β linked dimers, which exclusively exhibited type I photodynamic action by the generation of O_2_^−•^ after near-infrared irradiation [20]. Another way to prepare efficient long-wavelength type I and II photosensitizers involves the synthesis of sulfur-bridged BODIPY dimers [6]. Noteworthy, the unusual properties of BODIPY dimers can also be observed. Sukhanov et al. described orthogonal β-β BODIPY dimers with very low intersystem crossing efficiency and singlet oxygen generation resulting from the reversible exciton hopping between two BODIPY chromophores [21]. In contrast, Wang et al. described a non-orthogonal β,β-linked BODIPY dimer with the twisted orientation of two BODIPY units exhibiting red-shifted absorption and good efficiency of ROS generation [22]. Recently, intramolecular singlet fission (iSF) was proposed as another mechanism for triplet state generation in BODIPY dimers competing with SOCT-ISC. This process relies on generating a multiexcitonic state and diffusion of the formed triplet state into two states. The iSF process is probably not effective in orthogonal BODIPY dimers due to specific thermodynamic conditions but can play a role in the photophysical properties of non-orthogonal dimers [23,24].

Several examples of BODIPY dimers connected at *meso*-*meso* positions by different linkers have been reported. Dimers and trimers connected through nitrogen and oxygen atoms were prepared and studied as pH indicators and molecular rotors for optical and sensor applications [25]. Two BODIPY molecules connected by π-conjugated alkyne linkers revealed exceptional chemical and structural stability, indicating possible application in various device technologies and coatings [26]. Polycyclic *meso*-*meso* BODIPY dimer with a thiophene unit as a linker was prepared as a promising organic semiconducting material for optoelectronic applications [27]. Two BODIPY molecules connected by the *meso* disulfide linker can act as both donor and acceptor fluorescence resonance energy transfer (homo-FRET), and such dimer was used for imaging of biothiols for live-cell imaging of lysosomes [28]. Zou et al. synthesized the BODIPY dimer connected at *meso*-*meso* positions with a phenyl ring and its brominated and iodinated analogs. Monomeric dyes were also prepared for comparison. It was found that too many heavy atoms do not improve the singlet oxygen generation efficiently, but an increase in dark toxicity was observed [29]. Non-orthogonal *meso-meso* BODIPY dimers with furan bridge showed interesting spectroscopic properties. The dimer with all pyrrolic positions substituted with methyl and ethyl groups revealed a sterically hindered, twisted structure, resulting in the lack of significant spectral differences compared to the monomeric form. In contrast, less sterically hindered, more planar dimer with unsubstituted positions in the BODIPY core showed significantly red-shifted absorption and emission spectra [30]. The presented diversity of the structure and properties of BODIPY dimers and sometimes exhibited unexpected behavior encouraged us to examine these exceptional dyes further.

Liposomes are one of the drug carriers used to overcome common limitations of photodynamic agent candidates, such as high hydrophobicity, low solubility, a tendency to aggregate in an aqueous environment, poor bioavailability, or lack of targeting. As demonstrated by the case of verteporfin, the only registered photosensitizer in the liposomal form, liposomes are an effective system for delivering photosensitizers and ensuring good biocompatibility and biodegradability [31,32,33]. There are a few examples of liposome usage to improve the photodynamic effect of BODIPY dyes. The first report on PDT activity of liposomal formulations of BODIPYs given by Gayathari et al. concerns a series of styryl BODIPYs iodinated in the 2 and 6 positions. Liposomes circumvent the need to introduce hydrophilic functional groups to the BODIPY structure and provide high photocytotoxicity against the human ovarian carcinoma cell line (SK-OV-3) with the IC_50_ value of 0.35 μM for the most potent analog [34]. Styryl BODIPY derivative with cationic pyridinium groups was also incorporated into liposomes to improve bioavailability and internalization in cancer cells. After liposomal encapsulation, the IC_50_ values toward colorectal cancer (HCT-116) decreased from 3.95 to 0.81 μM [35]. Liposomal encapsulation of other styryl-BODIPYs with pyridine cations enhances their dispersibility and stability in a biological environment. Moreover, in vivo studies on murine mammary carcinoma (4T1) bearing mice proved that liposomes improved the accumulation and retention of BODIPY analog with two pyridine cations in tumors [36]. Chen et al. loaded BODIPY dimer into liposomes with anti-HIF antibodies for both hypoxia marker imaging and photodynamic therapy. The comparison of the free dimer with the liposome-encapsulated one showed improved solubility and higher selectivity of liposomal photosensitizer toward hypoxic tumor cells [37].

In this work, novel BODIPY dimers as potential photosensitizers are presented. Synthesis, characterization, photochemical properties, encapsulation in liposomes, as well as in vitro photodynamic anticancer and antibacterial studies are described.

## 2. Results and Discussion

### 2.1. Synthesis and Characterization

**Scheme 1:**
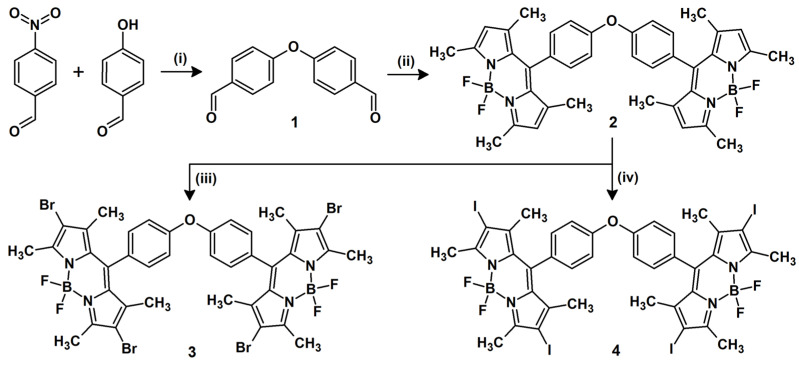
Synthesis of BODIPY dimers **2**–**4**. Reagents and conditions: (i) K_2_CO_3_, Pd/C, H_2_O, DMSO, 100 °C, 24 h; (ii) 1. 2,4-dimethylpyrrole, CF_3_COOH, CH_2_Cl_2_, 1 h; 2. DDQ, CH_2_Cl_2_, 1 h; 3. DIPEA, BF_3_*O(C_2_H_5_)_2_, 18 h, r.t.; (iii) N-bromosuccinimide, CH_2_Cl_2_, 30 min, r.t.; (iv) I_2_, HIO_3_, ethanol, 50 min, 70 °C.

The precursor of novel BODIPY dimers, 4,4′-oxydibenzaldehyde (4-(4-formylphenoxy)benzaldehyde, compound **1**), was synthesized using palladium on carbon (Pd/C) catalyzed cross-coupling reaction of 4-nitrobenzaldehyde and 4-hydroxybenzaldehyde according to Begum et al. procedure [38]. The subsequent reaction of obtained dialdehyde **1** with 2,4-dimethylpyrrole utilizing Galeotti et al. conditions [39] (three-step reaction in dichloromethane using trifluoroacetic acid, 2,3-dichloro-5,6-dicyano-1,4-benzoquinone (DDQ), *N*,*N*-diisopropylethylamine (DIPEA) and boron trifluoride diethyl etherate (BF_3_*O(C_2_H_5_)_2_)) led to novel BODIPY dimer **2** (Scheme 1). Noteworthy, 4,4′-oxydibenzaldehyde was previously used in Hantzsch and Biginelli reactions as a precursor of dihydropyridine dimers with promising antibacterial activity [40]. Next, BODIPY dimer **2** was reacted with N-bromosuccinimide (NBS) in dichloromethane generating the analog containing four bromine atoms (compound **3**). Similarly, iodinated derivative **4** was obtained in the reaction with iodine and iodic acid solution in ethanol (Scheme 1).

Mass spectrometry and various NMR techniques (^1^H NMR, ^13^C NMR, ^1^H-^1^H COSY, ^1^H-^13^C HSQC, ^1^H-^13^C HMBC) confirmed the structure of dimers **2**–**4**. In the aromatic region of the ^1^H NMR spectrum of dimer **2**, two doublets at 7.17 and 7.29 ppm from protons of the phenyl rings and a singlet at 6.01 ppm from pyrrolic protons were observed. In the aliphatic region, two singlets at 1.49 and 2.56 ppm, belonging to protons of the methyl groups, were found. In the ^1^H NMR spectra of the brominated and iodinated analogs **3** and **4**, no signal was observed at about 6 ppm, which was present in the spectrum of dimer **2** and was assigned to protons in positions 2 and 6 of the BODIPY core. This results from the substitution of the hydrogen atoms at these positions of dimer **2** with bromine or iodine atoms in the case of dimers **3** and **4**. ^13^C NMR spectra of **3** and **4** revealed eleven signals because carbon atoms from the methyl groups gave two separate signals at 13.7 and 14.0 ppm for brominated derivative **3**, and 16.1 and 17.3 ppm in the case of iodinated analog **4**. The other signals are analogous to the discussed earlier spectrum of compound **2**. A detailed analysis of the NMR spectra can be found in Supplementary Information.

### 2.2. Spectral Properties and Singlet Oxygen Generation

**Figure 2:**
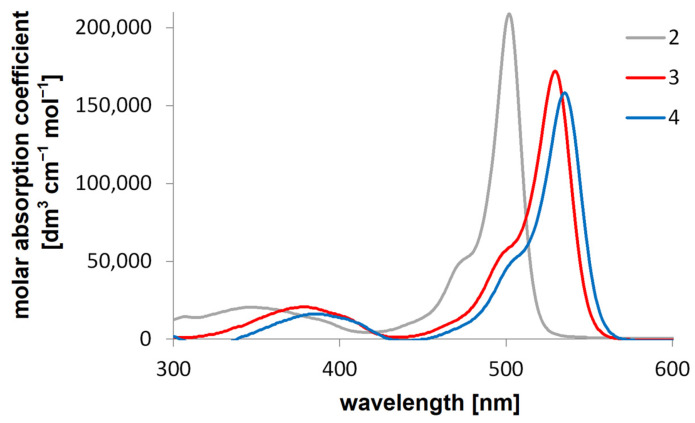
Absorption spectra of dimers **2**–**4** in dichloromethane.

Absorption properties of synthesized dimers **2**–**4** were determined by recording UV-Vis spectra in dichloromethane (DCM), methanol, ethanol, acetonitrile, *N*,*N*-dimethylformamide (DMF), and dimethyl sulfoxide (DMSO). The spectra of DCM are presented in Figure 2, and other solvents are shown in the Supplementary Information. These particular data, including absorption maxima (λ_Abs_) with logarithms of molar absorption coefficients (log ε) in DCM, are shown in the Supplementary Information (Table S4). It was found that the dimeric structure of compounds **2**–**4** did not significantly influence the positions of the absorption maxima, as they were very similar to those of monomeric BODIPY analogs containing *meso*-phenyl substituents [29,41,42]. A similar situation was observed in other non-orthogonal *meso-meso* dimeric BODIPYs [26,43]. The maximum absorption of compound **2** in the tested solvents was found between 498 and 502 nm, while in the range of 524–529 nm for brominated analog **3**, and from 530 to 536 nm in the case of iodinated dimer **4** (Table S4 in the Supplementary Information). The bathochromic shift in the absorption bands observed for halogenated dimers **3** and **4** results from the electron-accepting abilities of bromine and iodine atoms [29]. It is worth noting that BODIPY **2**–**4** revealed very high absorption intensity, as the values of molar coefficient (ε) for their absorption maxima were in the range of 5.05–5.33 (Table S4 in the Supplementary Information).

**Figure 3:**
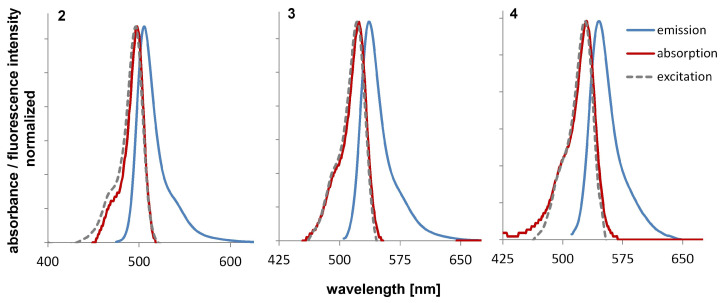
Long-wavelength absorption, excitation, and emission bands of dimers **2**–**4** in acetonitrile.

The emission properties of dimers **2**–**4** were determined in acetonitrile. The observed long-wavelength absorption, emission, and excitation bands are presented in Figure 3, whereas obtained data, including the wavelengths of emission maxima (λ_em_), Stokes shifts (Δλ), and fluorescence quantum yields (Φ_F_), are shown in Table 1.

The recorded emission spectra are the mirror images of the absorption ones, with small Stokes shifts and the maximum emission wavelength of 506, 537, and 545 nm for dimers **2**, **3**, and **4**, respectively (Figure 3, Table 1). The highest emission properties were found for dimer **2** with a fluorescence quantum yield of 0.69. Halogen-containing analogs exhibited much lower Φ_F_ values, equaling 0.25 for brominated analog **3** and 0.02 for iodinated derivative **4**, resulting from the heavy atom effect. The presence of atoms of a high atomic number in the BODIPY core significantly enhances the intersystem transition from singlet to triplet excited state, decreasing the fluorescence [41,44,45]. The fluorescence properties of dimers **2**–**4** can be compared with literature data of other fluorescent BODIPY dimers connected at *meso-meso* positions. Alamiry et al. measured the fluorescence quantum yield of the BODIPY dimer similar to dimer **2**, but additionally contained ethyl groups at the 2 and 6 positions, and obtained almost equal values of the fluorescence quantum yield (Φ_F_ = 0.7) [46]. Yahagh et al. prepared a monomeric and dimeric bis-styryl BODIPY derivative with a phenyl group at the *meso* position, with Φ_F_ values in toluene equaling 0.46 and 0.38 for the monomer and dimer, respectively [43]. The study of a series of BODIPY monomers, dimers, and trimers prepared by Li et al., revealed lower fluorescence intensity of dimers than those of monomeric forms, especially in polar solvents [47].

The relative chemical trapping method with Rose Bengal as a reference and 1,3-diphenylisobenzofuran (DPBF), which played the role of a singlet oxygen quencher, was used to calculate the quantum yields of singlet oxygen generation (Φ_Δ_) [48,49,50]. The results presented in Table 1 revealed a low singlet oxygen generation efficiency of dimer **2** with Φ_Δ_ value of only 0.02. On the other hand, the brominated and iodinated analogs revealed much higher efficiency with Φ_Δ_ of 0.45 for dimer **3** and 0.63 for **4**, resulting from the mentioned heavy atom effect. Bromine and iodine atoms in the BODIPY core enhance the spin–orbital coupling and intersystem crossing to the triplet state, from which photosensitizer can interact with oxygen to produce ^1^O_2_ [29,51,52]. The value for iodinated dimer **4** is even higher than the literature value for the referenced photosensitizer, Rose Bengal (Φ_Δ_ = 0.54 [53]).

### 2.3. Liposomal Formulations

**Figure 4:**
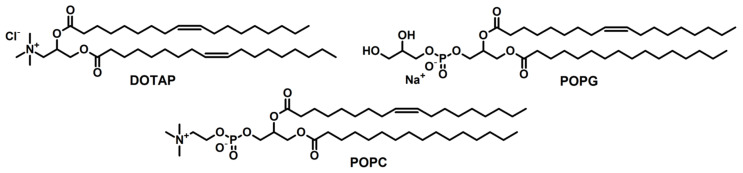
Structure of lipids used for liposome preparation.

The relatively large size and high lipophilicity of the obtained BODIPY dimers result in insolubility in a water environment, including culture media, which hinders the examination of their biological properties. For this reason, dimers **2**–**4** were incorporated into liposomes, which are known to be promising delivery systems for photosensitizers [31,54,55]. Two different liposomal formulations were prepared by a thin-film hydration method: (i) positively charged liposomes composed of 1,2-dioleoyl-3-trimethylammoniumpropane (DOTAP, chloride salt) and 1-palmitoyl-2-oleoyl-sn-glycero-3-phosphocholine (POPC); and (ii) negatively charged based on 1-palmitoyl-2-oleoyl-sn-glycero-3-phospho-(1′-rac-glycerol) (sodium salt, POPG) and POPC, were prepared by a thin-film hydration method [56,57,58,59] (Figure 4).

The measurements of extruded liposome size by the dynamic light scattering (DLS) method revealed that the z-average hydrodynamic size of particles ranges from 0.09 to 0.22 μm (Table 2). Electrophoretic light scattering (ELS) analysis revealed a positive charge on the surface of DOTAP:POPC liposomes with zeta potential in the range 43.0–56.2 mV and a negative charge in the case of POPG:POPC with zeta potential from −45.7 to −53.1 mV (Table 2).

### 2.4. In Vitro Photodynamic Inactivation of Planktonic Bacteria

The photodynamic activity of dimers **2**–**4** was evaluated in vitro on the representative strains of two main groups of bacteria—Gram-positive *Staphylococcus aureus* and Gram-negative *Escherichia coli*. The experiments were performed in light and dark conditions with liposomal formulations of tested dimers **2**–**4** at 5 and 1 µM concentrations. The viability of both bacteria strains was not affected by any liposomal formulations under dark conditions (Table 3 and Table 4). Upon exposure to light at 525 nm (total light dose of 7.2 J/cm^2^), positively charged DOTAP:POPC liposomes with brominated or iodinated dimers **3** and **4** revealed high photoinactivation activity against *S. aureus* at 5 µM concentration, with the log_10_ reduction in bacteria cells by 3.3 for liposomes containing brominated dimer **3** and 6.3 in the case of iodinated analog **4**. At a lower 1 µM concentration, the bacterial growth decreased by 2.0 and 2.2 for dimers **3** and **4**, respectively. Noteworthy, the American Society of Microbiology and the U.S. Food and Drug Administration (FDA) indicated that a 3 log_10_ reduction in cell counts must be reached to classify a new approach or agent as antibacterial or bactericidal [60,61]. Thus, only the values of log_10_ at a higher 5 µM concentration reached a satisfactory level. The DOTAP:POPC liposomes with dimer **2** did not show the photocytotoxic effect of both concentrations. Also, the negatively charged POPG:POPC liposomes with all tested dimers did not inactivate the *S. aureus* bacteria (Table 3).

Gram-negative bacteria such as *E. coli* are usually much less susceptible to photodynamic inactivation than Gram-positive bacteria due to the differences in the cell wall structure [62,63,64]. Nevertheless, in our studies, the activity of liposomal formulations of dimers **2**–**4** against Gram-negative *E. coli* was very similar to the described above activity toward *S. aureus*. Only DOTAP:POPC liposomes containing dimers **3** and **4** at 5 µM concentration revealed a high photocytotoxic effect on *E. coli* with log_10_ reduction of 3.5 and 6.1 for dimers **3** and **4**, respectively (Table 4).

The high activity of DOTAP:POPC liposomes against *E. coli* can result from the electrostatic interaction between the positively charged liposomes and negatively charged bacterial cell wall [52]. Previously examined photosensitizers incorporated into DOTAP:POPC liposomes, including oxospirochlorins, phthalocyanines, porphyrazines, and tribenzoporphyrazines, also revealed the promising photoinactivation of *S. aureus* and/or *E.coli*, but the concentration and the light dose required for their activity were significantly higher [65,66,67,68,69,70].

### 2.5. In Vitro Photodynamic Anticancer Activity

**Figure 5:**
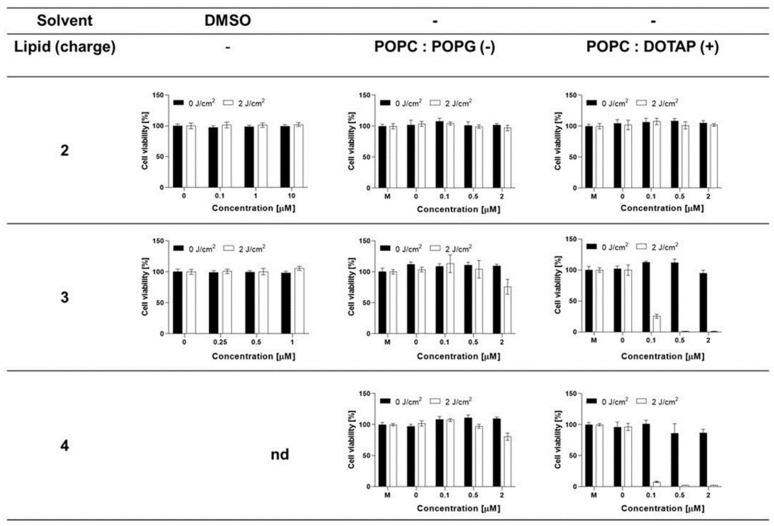
The activity of dimers **2**–**4** in free form (dissolved in DMSO and cell culture medium) and in positively and negatively charged liposomes towards MDA-MB-231 cells. The cells were treated with BODIPYs for 24 h and irradiated with a light dose of 2 J/cm^2^ or not irradiated in the case of the dark control. The MTT assay was performed 24 h post-irradiation. The data presented results from preliminary experiments. Abbreviations: M—cell culture medium, nd—not determined.

In the preliminary experiments, BODIPY dimers **2**–**4** were dissolved in DMSO and diluted to the desired concentrations in a cell culture medium. BODIPY **2** was tested at 10, 1, and 0.1 µM concentrations. Due to the limited solubility of BODIPY **3** in DMSO, the concentration range was reduced to 1, 0.5, and 0.25 µM. BODIPY **4** could not be dissolved in the same way to achieve a concentration of 1 mM; therefore, the cytotoxicity of this compound was not determined. The MTT assay showed that none of the tested BODIPYs significantly affected cell viability (Figure 5).

Due to the observed solubility issues, liposomal formulations of the tested compounds were prepared in the next stage of the research. For this purpose, liposomes with both positive and negative charges were prepared, and the experiments were conducted on the MDA-MB-231 cell line. The surface charge on lipids (negative or positive) can decrease the tendency to aggregation and increase the liposome–cell interaction compared to neutral liposomes [71]; thus, these two liposomal formulations were selected in our study. The highest concentrations of the liposomal formulations were selected based on our previous experience with them, where we found that empty liposomes can exhibit cytotoxicity on their own. Since the cell membrane is negatively charged, cationic liposomes can increase cellular uptake compared to negatively charged and neutral liposomes [72]. Sakai-Kato reported that cationic liposomes are internalized at larger sizes than other liposomes and are more sensitive to liposome diameter [73].

**Figure 6:**
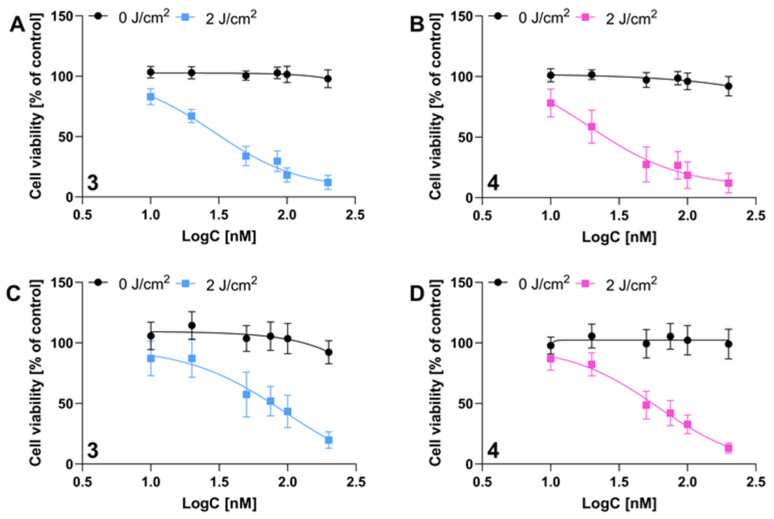
Dose–response curves plotted for BODIPYs **3** and **4** against MDA-MB-231 and A2780 cells. Panels (**A**) and (**B**) show the viability of MDA-MB-231 cells for BODIPYs **3** and **4**, respectively. Panels (**C**) and (**D**) show the viability of A2780 cells for BODIPYs **3** and **4**, respectively. Cell viability was measured using the MTT assay 24 h after irradiation (phototoxicity) or without irradiation (dark toxicity). Cell culture medium and empty liposomal formulations were used as controls. Data are presented as mean values ± SD, calculated from five independent experiments (biological replicates) performed in six technical replicates for MDA-MB-231 cells and four independent experiments (biological replicates) performed in six technical replicates for A2780 cells.

Interestingly, as presented in Figure 5, only positive charged liposomal formulations for BODIPYs **3** and **4** were active. The cationic liposomes can pass the cell membrane via an endocytic pathway and membrane fusion. It was reported that membrane fusion could deliver the encapsulated compound directly to the cytoplasm and overcome endosomal/lysosomal degradation. Thus, the cationic liposomes may not only improve the cargo transport by a cellular membrane but also, if the membrane fusion pathway is involved in this process, the loaded compound can be protected from endosomal and metabolic degradations, which can finally increase efficacy [74]. Several studies showed that DOTAP, as a cationic lipid in the liposomal formulation, could enhance fusogenicity [75,76,77]. On the other hand, negatively charged liposomes were found to be less stable than positively charged liposomes and more easily degraded by the reticuloendothelial system [71]. The presented characteristics of positively and negatively charged liposomes could have contributed to our results, showing biological activity only for the positively charged liposomes. In the liposomal formulations, we tested three compounds, but only two proved to be active, which may be related to the well-known heavy atom effect in BODIPY molecules [78]. BODIPY **3** and **4** are brominated and iodinated dimers, respectively, with a significantly higher singlet oxygen generation than BODIPY **2** without heavy atoms. Since singlet oxygen is one of the main ROS generated during PDT, the higher generation of singlet oxygen by BODIPYs **3** and **4** likely translates into a more substantial effect. Furthermore, these results are consistent with the data obtained for compound **2** in microbiological studies, where no photodynamic activity was observed either. Further experiments were performed for the positively charged liposomal formulation of **3** and **4** towards MDA-MB-231 and A2780 cells to determine the IC_50_ values. The dose–response curves are presented in Figure 6.

As shown in Table 5, both BODIPYs affected cell viability at nanomolar concentrations. Noteworthy, the BODIPYs **3** and **4** showed similar cytotoxic activity against the cell lines on which they were tested. BODIPYs **3** and **4** in positively charged liposomes did not show dark toxicity at the tested concentration range.

BODIPYs **3** and **4** were slightly more active against triple-negative breast cancer cells than ovarian cancer cells, with IC_50_ values of 34 and 29 nM for **3** and **4**, respectively. This is particularly important since the MDA-MB-231 cell line represents triple-negative breast cancer (TNBC), which has limited therapeutic options. Since TNBC is defined by the absence of estrogen receptors (ER), progesterone receptors (PR), and human epidermal growth factor receptor 2 (HER2), it does not respond to hormonal therapy or conventional immunotherapy and is resistant to chemotherapy [79].

Noteworthy, the photodynamic activity of BODIPYs **3** and **4** with IC_50_ values in the 29–76 nM range can be considered very high. Epelde-Elezcano and co-workers also observed the activity in the nanomolar concentration range for the *meso*-β orthogonal dimer, which revealed high phototoxicity on HeLa cells even at 50 nM concentration [14]. Guo et al. synthesized three *meso*-α-linked BODIPY dimers and examined their in vitro photodynamic activity on Hela cells. Two dimers revealed high phototoxicity with IC_50_ values of 259 and 207 nM [19]. Zou et al. prepared *meso-meso* BODIPY dimer and its brominated and iodinated analogs. The cytotoxic activity was tested on HeLa cells after preparing hydrophilic nanoparticles using the re-precipitation method. The calculated IC_50_ values were equaled 3.3 and 2.8 μM for nanoparticles of brominated and iodinated BODIPY dimers, respectively [29].

## 3. Materials and Methods

### 3.1. Instrumentation and General Procedures

The Heidolph MR Hei-Tec magnetic stirrers, Radleys Findenser air condensers, heat on heating blocks, argon atmosphere, Erbauer heat gun, and glassware were used to conduct chemical reactions. The SiliCycle plates with F-254 indicator visualized with UV illumination (λ_max_ 254 or 365 nm) were utilized for thin layer chromatography (TLC). The Buchi R-100 and Heidolph Hei-VAP rotary evaporators were used for solvent removal at reduced pressure. The Shimadzu UV-1900i spectrophotometer was used to record UV-Vis spectra. The Bruker AvanceCore 400 spectrometer at the Center of Innovative Pharmaceutical Technology at Poznan University of Medical Sciences was utilized for NMR spectra recording at 400.13 MHz for ^1^H and 100.61 MHz for ^13^C NMR. Chemical shifts (δ) are referred to as a residual solvent peak and quoted in parts per million (ppm). The s and d abbreviations were used for singlet and doublet signals, respectively. The High-Resolution Bruker QTOF Impact HD spectrometer at the Center for Advanced Technologies in Poznan (Poland) was used for mass spectra (HRMS) measurements. The *m*/*z* values were given together with the intensity as a percentage of the maximal value and the difference between the calculated and found masses in ppm (Δ ppm).

### 3.2. Synthetic Procedures

Compound **1** (4,4′-oxydibenzaldehyde) was synthesized following the Begum et al. procedure [38].

#### 3.2.1. Compound **2** (dimer-H)

4,4′-Oxydibenzaldehyde (compound **1**, 848 mg, 3.75 mmol) and 2,4-dimethylpyrrole (1.55 mL, 15.0 mmol) were dissolved in dichloromethane (200 mL) with the addition of two drops of trifluoroacetic acid. Then, a solution of 2,3-dichloro-5,6-dicyano-1,4-benzoquinone (DDQ, 3.4 g, 15.0 mmol) in dichloromethane (100 mL) was added, and the reaction was continued for another hour. Next, *N*,*N*-diisopropylethylamine (19.6 mL, 112 mmol) was added, and BF_3_*O(C_2_H_5_)_2_ (18.5 mL, 150 mmol) was added dropwise within a few minutes. After the reaction was completed (18 h), the resulting mixture was washed with water and filtered through Celite. The obtained mixture was extracted with water and dichloromethane, and the organic layer was dried with anhydrous Na_2_SO_4_ and evaporated. The solid residue was purified by column chromatography using dichloromethane and hexane in a 1:1 ratio (*v*/*v*) as the mobile phase. The crystallization from dichloromethane using n-pentane gave an orange solid of compound **2** (504 mg, 20%). R_f_ = 0.31 (dichloromethane-hexane, 2:1); UV-Vis (dichloromethane) λ_max_, nm (log ε) 347 (4.31), 502 (5.32). ^1^H NMR (400.1 MHz; CDCl_3_) δ 7.29 (d, 4H, ^3^*J =* 9 Hz, ArH), 7.17 (d, 4H, ^3^*J =* 9 Hz, ArH), 6.01 (s, 4H, ArH), 2.56 (s, 12H, -CH_3_), 1.49 (s, 12H, -CH_3_). ^13^C NMR (100.6 MHz; CDCl_3_) δ 157.5, 155.8, 143.0, 141.0, 131.7, 130.5, 130.0, 121.5, 119.6, 14.7. HRMS (ESI) *m*/*z* calcd. for C_38_H_35_B_2_F_4_N_4_O [M-H]^−^ 661.2933, found 661.2941—100%, (Δ = 1.2 ppm).

#### 3.2.2. Compound **3** (dimer-Br)

Compound **2** (166 mg; 0.25 mmol) was dissolved in dichloromethane (50 mL), and the solution of N-bromosuccinimide (267 mg; 1.5 mmol) in dichloromethane (10 mL) was added dropwise. The reaction mixture was stirred for 30 min at room temperature, and the solvent was evaporated. The solid residue was chromatographed starting with hexane and dichloromethane in a 2:1 ratio (*v*/*v*) as the mobile phase, then the mixture in a 1:1 ratio was used, and the separation was finished using dichloromethane alone. The precipitate of compound **3** was obtained from a dichloromethane and n-pentane mixture (121 mg, 48%). R_f_ = 0.40 (dichloromethane-hexane, 1:1); UV-Vis (dichloromethane) λ_max_, nm (log ε) 378 (4.32), 529 (5.24). ^1^H NMR (400.1 MHz; CDCl_3_) δ 7.29 (d, 4H, ^3^*J =* 9 Hz, ArH), 7.21 (d, 4H, ^3^*J =* 9 Hz, ArH), 2.62 (s, 12H, -CH_3_), 1.50 (s, 12H, -CH_3_). ^13^C NMR (100.6 MHz; CDCl_3_) δ 157.6, 154.3, 141.1, 140.4, 130.6, 129.9, 119.8, 112.0, 14.0, 13.7. HRMS (ESI) *m*/*z* calcd. for C_38_H_31_B_2_Br_4_F_4_N_4_O [M-H]^−^ 972.9354, found 972.9358—19.1%, (Δ = 0.4 ppm).

#### 3.2.3. Compound **4** (dimer-I)

Compound **3** (66 mg; 0.1 mmol) and iodine (76 mg; 0.3 mmol) were dissolved in ethanol (20 mL). Next, a solution of iodic acid (70 mg; 0.4 mmol) in 0.8 mL of purified water was added, and the reaction was carried out for 50 min at 70 °C. The reaction mixture was cooled to room temperature, and the solid residue was filtrated off and chromatographed (from hexane-dichloromethane 2:1 (*v*/*v*)) to dichloromethane. The precipitate of compound **4** was obtained from a dichloromethane and n-pentane mixture (78 mg, 85%). R_f_ = 0.38 (dichloromethane-hexane, 1:1); UV-Vis (dichloromethane) λ_max_, nm (log ε) 386 (4.21), 535 (5.20). ^1^H NMR (400.1 MHz; CDCl_3_) δ 7.28 (d, 4H, ^3^*J =* 9 Hz, ArH), 7.21 (d, 4H, ^3^*J =* 9 Hz, ArH), 2.66 (s, 12H, -CH_3_), 1.51 (s, 12H, -CH_3_). ^13^C NMR (100.6 MHz; CDCl3) δ 157.6, 157.1, 145.1, 140.4, 131.5, 130.2, 129.9, 119.8, 85.9, 17.3, 16.1. HRMS (ESI) *m*/*z* calcd. for C_38_H_31_B_2_F_4_I_4_N_4_O [M-H]^−^ 1164.8799, found 1164.8765– 100.0% (Δ = 2.9 ppm).

### 3.3. Absorption and Emission Properties

The UV-Vis absorption measurements were conducted in 10 mm Hellma quartz cuvettes using a Shimadzu UV-1900i spectrophotometer (Shimadzu Deutschland, Duisburg, Germany). The fluorescence measurements were executed in acetonitrile solutions using a Jasco 6200 spectrofluorimeter [48,80,81,82]. Fluorescein solution in 0.1 M NaOH (Φ_Fref_ = 0.92 [83]) was used as a reference for dimer **2**, and Rhodamine 6G in ethanol was used as a reference for dimers **3** and **4** (Φ_Fref_ = 0.95 [83]). The solutions of BODIPYs **2**–**4** and a reference, with an absorbance at the maximum absorption set to 0.05, were excited at 475 nm for dimer **2** and 505 nm for **3** and **4.** For the fluorescence quantum yield (Φ_F_) calculations, the following equation was used:

$$\phi_{F\;}=\phi_{F\;ref.\;}\;\frac{F_{sample}}{F_{ref.}}\;\frac{A_{ref.}}{A_{sample}}\;\frac{\eta_{sample}^{2}}{\eta_{ref.}^{2}}$$

The area under the emission curve is represented by F; the absorbance at the excitation wavelength is represented by A; the solvent refractive index [84,85] is represented by *η*.

### 3.4. Photosensitized Production of Singlet Oxygen

The relative chemical trapping method with Rose Bengal (Aldrich) as a reference and 1,3-diphenylisobenzofuran (DPBF, Aldrich) as a singlet oxygen quencher was used to determine the quantum yields of singlet oxygen generation (Φ_Δ_) [48,49,50]. The acetonitrile solutions of dimers **2**–**4** and Rose Bengal containing DPBF were prepared and irradiated in 10 mm Hellma quartz cuvettes, while the UV-Vis spectra were recorded at specific time intervals. The wavelength of light from a 150 W high-pressure Xe lamp and M250/1200/U monochromator (Optel, Opole Poland) was set to 510 nm, and its intensity measured by ThorLabs PM100D power and energy meter with the S120VC photodiode sensor equaled 0.5 mW/cm^2^. Three measurements for each photosensitizer were performed and used to calculate Φ_Δ_ values according to the equation:

$$\phi_{\Delta }=\phi_{∆\;ref.}\frac{k_{sample}}{k_{ref.}}\frac{{1-10}^{{-A}_{ref.}}}{{1-10}^{{-A}_{sample}}}$$

The quantum yield of singlet oxygen generation is represented by Φ_Δ_ (Φ_Δ ref_ = 0.54 [53]); the constant rate of light-induced DPBF decomposition is represented by k; the absorption correction factor is 1–10^−A^, with A referring to the absorbance at the irradiation wavelength.

### 3.5. Liposome Preparation

Liposomal formulations containing dimers **2**–**4** were prepared based on the following lipids: 1,2-dioleoyl-3-trimethylammoniumpropane (DOTAP, chloride salt, Lipoid, Ludwigshafen, Germany), 1-palmitoyl-2-oleoyl-sn-glycero-3-phospho-(1′-rac-glycerol) (sodium salt, POPG, Avanti Polar Lipids, Alabaster, AL, USA), and 1-palmitoyl-2-oleoyl-sn-glycero-3-phosphocholine (POPC, Lipoid). Two types of liposomes, positively charged composed of DOTAP and POPC and negatively charged based on POPG and POPC, were prepared by the thin-film hydration method [56,57,58,59]. The measured volumes of the lipid stock solutions in chloroform (25 mg/mL) and dimers (0.4 mg/mL), were placed in glass tubes, mixed, and rotary evaporated to dryness. The lipid films obtained in the glass tubes were dried in a vacuum to evaporate any remaining chloroform. Next, the phosphate-buffered saline (PBS, pH 7.4) was added, and the mixture was dispersed by vortexing and sonication for 5–10 min. Next, a syringe extruder (Avanti Polar Lipids) was used to repeatedly push the obtained liposome suspensions (15 times) through the 0.4 um pore polycarbonate membranes (Whatman, Maidstone, UK) to unify the size distribution. In addition, the liposomes without dimers as controls were prepared the same way. All liposomal formulations were kept in the dark at 4 °C. The liposome parameters, including the size expressed as Z-average, polydispersity index (PDI), and zeta potential (ζ) were determined using Malvern Zetasizer Pro (Malvern Panalytical, Malvern, UK).

### 3.6. In Vitro Photodynamic Inactivation of Planktonic Bacteria

The American Type Culture Collection bacteria Staphylococcus aureus ATCC 29213 and Escherichia coli ATCC 25922 were utilized for the microbiologic studies conducted following the previously elaborated procedures [69,78,86]. Liposomal formulations of dimers **2**–**4** were diluted in sterile water to obtain the desired concentrations. The prepared solutions were transferred to a 96-well titration plate to which microbial suspensions (150 µL, 2.0 × 10^5^ CFU) were added. Control solutions containing only microbial suspensions were also prepared. Two titration plates were prepared for each experiment and preincubated in the dark for 20 min. Next, one plate was kept in the dark as the control without exposure to light. The second one was irradiated with light of 525 nm using the High-Power LED MultiChip Emitters (RoithnerLaserTechnik, Vienna, Austria) for 30 min. The light intensity at the surface of the plate was measured by Thorlabs PM100D power (Thorlabs GmbH, Bergkirchen, Germany) and energy meter with an S130C slim photodiode sensor (Thorlabs GmbH, Bergkirchen, Germany) and equaled 4 mW/cm^2^ (total light dose of 7.2 J/cm^2^). The viability of bacteria was determined by the agar plate method and presented as a log_10_ reduction in the number of bacteria. The number of bacteria in controls without photosensitizers was 6.8 ± 0.2 log_10_ for *S. aureus* and 6.2 ± 0.3 log_10_ for *E. coli*.

### 3.7. Cytotoxic Activity

#### 3.7.1. Materials

Reagents used for in vitro experiments, such as Dulbecco’s Modified Eagle Medium (DMEM), fetal bovine serum (FBS), Dulbecco’s Modified Phosphate-Buffered Saline (DPBS), trypsin-EDTA, L-glutamine, penicillin and streptomycin solution, dimethyl sulfoxide (DMSO), and 3-(4,5-dimethylthiazol-2-yl)-2,5-diphenyltetrazolium bromide (MTT), were obtained from Sigma-Aldrich (St. Louis, MO, USA). The DMSO used for dissolving formazan crystals was obtained from Avantor Performance Materials (Gliwice, Poland)

#### 3.7.2. Cell Culture

The human ovarian carcinoma cell line (A2780) and human breast adenocarcinoma (MDA-MB-231) were purchased from the European Collection of Authenticated Cell Cultures (ECACC, Salisbury, UK) and American Type Culture Collection (ATCC, Manassas, VA, USA), respectively. Both cell lines were cultured in DMEM supplemented with 10% (*v*/*v*) fetal bovine serum, 1% (*v*/*v*) L- glutamine (200 mM), and 1% (*v*/*v*) 10,000 units/mL penicillin, and 10 mg/mL streptomycin solution.

#### 3.7.3. Preliminary Experiments: The Cytotoxic Activity of Free Compounds Dissolved in DMSO

MDA-MB-231 cells at a density of 15,000 cells per well, were seeded into a 96-well plate and incubated overnight under standard cell culture conditions. The cells were treated with compounds **2** and **3** at concentrations of 10, 1, and 0.1 µM, and 1, 0.5, and 0.25 µM, respectively. DMSO was used as a control at a concentration of 0.1% in the cell culture medium. The cells were exposed to the tested BODIPYs for 24 h. Afterward, the solution was removed, the cells were washed twice with PBS, and a fresh medium was added to the wells. The cells were irradiated with 525 nm light using High-Power LED MultiChip Emitters (Roithner LaserTechnik, Vienna, Austria) at a light dose of 2 J/cm^2^. A PM16-130 power meter with a slim photodiode sensor (ThorLabs, Newton, NJ, USA) was used to measure light power before each experiment. Following irradiation, the cells were incubated for an additional 24 h, after which the MTT assay was performed. The MTT solution (stock concentration of 5 mg/mL in PBS) was mixed with the cell culture medium at a ratio of 20 µL to 150 µL (final concentration of 0.59 mg/mL) and incubated for 1.5 h under standard cell culture conditions. Subsequently, the MTT solution was removed, and 200 µL of DMSO was added to each well. Absorbance was measured using a plate reader (Biotek Instruments, Elx-800, Winooski, VT, USA) at a wavelength of 570 nm.

#### 3.7.4. Preliminary Experiments: The Cytotoxic Activity of Liposomal Formulations of Compounds **2**–**4**

The MDA-MB-231 cells were seeded at a density of 15,000 cells/well. After overnight incubation, liposomal formulations of compounds **2**–**4** were added to the cells at concentrations of 2, 1, and 0.1 µM in a cell culture medium. Two negative controls were prepared: cell culture medium and cell culture medium with empty liposomes at a concentration corresponding to the highest tested concentration. The cells were irradiated with 505 nm and 525 nm light for BODIPY **2** and 525 nm for BODIPYs **3** and **4** at a light dose of 2 J/cm^2^. The MTT assay was performed as described above.

#### 3.7.5. Cytotoxic Effect of Liposomes DOTAP:POPC Encapsulating BODIPYs **3** and **4**

A2780 and MDA-MB-231 cells were seeded at a density of 15,000 cells per well and incubated overnight. Liposomal formulations of compounds **3** and **4** were added to the cells at concentrations of 200, 100, 75, 50, 20, and 10 nM. Two negative controls were prepared: cell culture medium and cell culture medium with empty liposomes at a concentration corresponding to the highest tested concentration. After 24 h of incubation, the cells were irradiated with light at 525 nm (light dose of 2 J/cm^2^). Twenty-four hours post-irradiation, the MTT assay was performed as described above.

The IC_50_ values were calculated using GraphPad Prism 8 software (GraphPad Software, Inc., La Jolla, CA, USA). A variable slope (four-parameter) analysis was used to calculate dose–response curves and determine IC_50_ values. To calculate the IC_50_ values, four biological replicates (each performed with six technical replicates) were conducted for A2780 cells and five biological replicates for MDA-MB-231 cells.

## 4. Conclusions

Novel BODIPY dimer connected at *meso-meso* positions and its brominated and iodinated analogs were synthesized and characterized using mass spectrometry (HRMS) and various 1D and 2D NMR techniques. Absorption properties investigated in different organic solvents revealed an intensive absorption band between 498 and 502 nm for dimer **2** unsubstituted at 2 and 6 positions and in the range of 524–536 nm for brominated and iodinated analogs **3** and **4**. Noteworthy, the dimeric structure of compounds **2**–**4** did not significantly influence the positions of the absorption maxima. The emission spectra resemble the mirror images of the absorption ones, with small Stokes shifts. The highest fluorescence quantum yield was found for dimer 2 (Φ_F_ = 0.69). In contrast, halogen-containing analogs revealed much lower fluorescent intensity with Φ_F_ values of 0.25 for brominated dimer **3** and 0.02 for the iodinated analog **4**, which results from the heavy atom effect. The quantum yields of singlet oxygen generation follow the opposite order, with the highest Φ_Δ_ value of 0.63 for iodinated derivative **4** and the lowest for dimer **2** (Φ_Δ_ = 0.02). For the in vitro photodynamic activity studies, BODIPY dimers **2**–**4** were incorporated into two different types of liposomes, positively charged composed of DOTAP and POPC and negatively charged based on POPG and POPC. The photoinactivation studies on Gram-positive bacteria *Staphylococcus aureus* revealed high activity of brominated and iodinated dimers incorporated into the positively charged DOTAP:POPC liposomes with the reduction in bacteria cells by 3.3 log_10_ for dimer **3** and 6.3 log_10_ in the case of analog **4**, at 5 µM concentration. The DOTAP:POPC liposomes with dimer **2** and the negatively charged POPG:POPC liposomes with all tested compounds did not show the photocytotoxic effect. The activity of liposomes with dimers **2**–**4** against Gram-negative *E. coli* was very similar to the activity toward *S. aureus*. Only DOTAP:POPC liposomes containing dimers **3** and **4** at 5 µM concentration revealed a high photocytotoxic effect with log_10_ reduction of 3.5 and 6.1 for dimers **3** and **4**, respectively. Anticancer studies on human breast adenocarcinoma MDA-MB-231 and human ovarian carcinoma A2780 cells revealed that only positive charged liposomal formulations containing BODIPYs **3** and **4** were active. BODIPYs **3** and **4** affected cell viability at nanomolar concentration and showed similar cytotoxic activity against both cell lines. They were more active against MDA-MB-231 cells with IC_50_ values of 34 and 29 nM for **3** and **4**, respectively, whereas the IC_50_ values for A2780 cells equaled 76 and 57 nM. This is particularly important since the MDA-MB-231 cell line represents triple-negative breast cancer, which has limited therapeutic options.

## Figures and Tables

**Table 1:** UV-Vis absorption maxima (λ_Abs_), logarithms of molar absorption coefficients (log ε), emission wavelengths (λ_em_), Stokes shifts (Δλ), quantum yields of fluorescence (Φ_F_), and singlet oxygen generation yields (Φ_Δ_) of dimers **2**–**4** in acetonitrile.

BODIPY	λ_Abs_[nm]	log ε	λ_em_ [nm]	Δλ [nm]	Φ_F_	Φ_Δ_
**2**	498	5.27	506	8	0.69	0.02
**3**	524	5.21	537	13	0.25	0.45
**4**	530	5.05	545	15	0.02	0.63

**Table 2:** Characteristics of liposomal formulations with dimers **2**–**4**.

Liposomal Composition	Molar Ratios	Z-Average ± SD [μm]	Polidispersity Index ± SD	Zeta Potential ± SD [mV]
DOTAP:POPC	2:8	0.22 ± 0.01	0.23 ± 0.01	56.2 ± 5.2
**2**:DOTAP:POPC	0.1:2:8	0.13 ± 0.02	0.18 ± 0.05	43.0 ± 1.2
**3**:DOTAP:POPC	0.1:2:8	0.17 ± 0.03	0.23 ± 0.03	52.0 ± 5.9
**4**:DOTAP:POPC	0.1:2:8	0.16 ± 0.03	0.22 ± 0.06	49.5 ± 4.9
POPG:POPC	2:8	0.22 ± 0.02	0.29 ± 0.08	−53.1 ± 1.5
**2**:POPG:POPC	0.1:2:8	0.12 ± 0.01	0.17 ± 0.02	−52.3 ± 1.2
**3**:POPG:POPC	0.1:2:8	0.16 ± 0.04	0.23 ± 0.01	−50.1 ± 2.3
**4**:POPG:POPC	0.1:2:8	0.09 ± 0.01	0.14 ± 0.03	−45.7 ± 2.8

**Table 3:** Log_10_ reductions in *S. aureus* (ATCC 25923) treated with dimers **2**–**4** in liposomal formulations.

Log_10_ Reduction in *S. aureus* Bacteria					
		DOTAP:POPC		POPG:POPC	
BODIPY	C [µM]	Light	Dark	Light	Dark
**2**	5.0	0.1 ± 0.3	0.0 ± 0.1	0.3 ± 0.2	0.1 ± 0.1
	1.0	0.1 ± 0.3	0.1 ± 0.1	0.2 ± 0.1	0.0 ± 0.2
**3**	5.0	3.3 ± 0.4	0.0 ± 0.1	0.5 ± 0.5	0.1 ± 0.3
	1.0	2.0 ± 0.4	0.0 ± 0.1	0.3 ± 0.5	0.0 ± 0.1
**4**	5.0	6.3 ± 0.6	0.1 ± 0.1	0.5 ± 0.1	0.1 ± 0.1
	1.0	2.2 ± 0.3	−0.1 ± 0.5	0.1 ± 0.1	0.1 ± 0.2

Data represent the means ± standard deviation obtained from three independent experiments performed in duplicate.

**Table 4:** Log_10_ reductions in *E. coli* (ATCC 25922) treated with dimers **2**–**4** in liposomal formulations.

Log_10_ Reduction in *E. coli* Bacteria					
		DOTAP:POPC		POPG:POPC	
BODIPY	C [µM]	Light	Dark	Light	Dark
**2**	5.0	0.3 ± 0.2	0.1 ± 0.2	0.0 ± 0.1	−0.2 ± 0.1
	1.0	0.3 ± 0.2	0.2 ± 0.2	−0.1 ± 0.2	−0.2 ± 0.1
**3**	5.0	3.5 ± 0.3	0.2 ± 0.3	0.0 ± 0.1	0.0 ± 0.1
	1.0	2.4 ± 0.2	0.1 ± 0.2	0.0 ± 0.1	−0.1 ± 0.1
**4**	5.0	6.1 ± 0.3	−0.1 ± 0.2	0.6 ± 0.2	0.1 ± 0.1
	1.0	2.5 ± 0.2	0.2 ± 0.2	0.2 ± 0.2	0.1 ± 0.2

Data represent the means ± standard deviation obtained from three independent experiments performed in duplicate.

**Table 5:** The IC_50_ values of BODIPYs **3** and **4** in liposomal DOTAP:POPC formulation. The data represent the mean values and standard deviations from five independent experiments for MDA-MB-231 cells and four experiments for A2780 cells.

IC_50_ Values [nM]				
	MDA-MB-231		A2780	
BODIPY	0 J/cm^2^	2 J/cm^2^	0 J/cm^2^	2 J/cm^2^
**3**	>200	34.18 ± 8.30	>200	76.31 ± 35.93
**4**	>200	29.38 ± 13.03	>200	57.26 ± 14.48

## Data Availability

The original contributions presented in the study are included in the article/Supplementary Material, further inquiries can be directed to the corresponding author.

## Supplementary Materials

The following supporting information can be downloaded at: https://www.mdpi.com/article/10.3390/molecules29225304/s1. Figure S1: ^1^H NMR of **2**; Figure S2: ^13^C NMR of **2**; Figure S3: ^1^H and (^13^C) chemical shift values [ppm] and key correlations observed in 2D NMR spectra of **2**; Figure S4: ^1^H NMR of **3**; Figure S5: ^13^C NMR of **3**; Figure S6: ^1^H and (^13^C) chemical shift values [ppm] and key correlations observed in 2D NMR spectra of **3**; Figure S7:^1^H NMR of **4**; Figure S8: ^13^C NMR of **4**; Figure S9: ^1^H and (^13^C) chemical shift values [ppm] and key correlations observed in 2D NMR spectra of **4**; Figure S10: HRMS spectrum of **2**; Figure S11: HRMS spectra of **3**; Figure S12: HRMS spectrum of **4**; Figure S13: Absorption spectra of compounds **2**–**4** in methanol; Figure S14: Absorption spectra of compounds **2**–**4** in ethanol; Figure S15: Absorption spectra of compounds **2**–**4** in DMF; Figure S16: Absorption spectra of compounds 2–4 in DMSO; Figure S17: Absorption spectra of compounds **2**–**4** in acetonitrile; Table S1: NMR data for **2**; Table S2: NMR data for **3**; Table S3: NMR data for **4**; Table S4: UV-Vis absorption maxima (λAbs) and logarithms of molar absorption coefficients (logε) of compounds **2**–**4** in various solvent.
