# Supplementary material for: Liposomal Formulations of Novel BODIPY Dimers as Promising Photosensitizers for Antibacterial and Anticancer Treatment
Source: Molecules. 2024 Nov 10;29(22):5304. doi: 10.3390/molecules29225304 (PMC11596046; doi:10.3390/molecules29225304)
Supplement: Supplementary file 1 [file molecules-29-05304-s001.zip › molecules-3240463-supplementary.pdf]

Liposomal formulations of novel BODIPY dimers as promising photosensitizers for antibacterial and anticancer treatment

Weronika Porolnik[1,2,3], Magdalena Ratajczak[4], Aleksandra Mackowiak[1], Marek Murias[2], Malgorzata Kucinska[2] and Jaroslaw Piskorz[1*]

[1] Chair and Department of Inorganic and Analytical Chemistry, Poznan University of Medical Sciences, Rokietnicka 3, 60-806 Poznan.

[2] Chair and Department of Toxicology, Poznan University of Medical Sciences, Rokietnicka 3, 60-806 Poznan, Poland.

[3] Doctoral School, Poznan University of Medical Sciences, Bukowska 70, 60-812 Poznan, Poland.

[4] Chair and Department of Genetics and Pharmaceutical Microbiology, Poznan University of Medical Sciences, Rokietnicka 3, 60-806 Poznan, Poland.

* Correspondence: e-mail: piskorzj@ump.edu.pl; Tel.: +48 61 6418341.

Supplementary Information

Table of Contents

Fig. S1. ^{1}H NMR of **2** ……………………………………………………………3
Fig. S2. ^{13}C NMR of **2** ……………………………………………………………3
Table S1. NMR data for **2...**……………………………………………………………4
Fig. S3. ^{1}H and (^{13}C) chemical shift values [ppm] and key correlations observed in 2D NMR spectra of **2**……………………………………………………………4
Fig. S4. ^{1}H NMR of **3** ……………………………………………………………5
Fig. S5. ^{13}C NMR of **3**……………………………………………………………5
Table S2. NMR data for **3**……………………………………………………………6
Fig. S6. ^{1}H and (^{13}C) chemical shift values [ppm] and key correlations observed in 2D NMR spectra of **3**……………………………………………………………6
Fig. S7. ^{1}H NMR of **4**……………………………………………………………7
Fig. S8. ^{13}C NMR of **4**……………………………………………………………7
Table S3. NMR data for **4** ……………………………………………………………8
Fig. S9. ^{1}H and (^{13}C) chemical shift values [ppm] and key correlations observed in 2D NMR spectra of **4**……………………………………………………………8
Fig. S10. HRMS spectrum of **2**……………………………………………………………9
Fig. S11. HRMS spectra of **3** ……………………………………………………………10
Fig. S12. HRMS spectrum of **4** ……………………………………………………………12
Fig. S13. Absorption spectra of compounds **2-4** in methanol……………………………13
Fig. S14. Absorption spectra of compounds **2-4** in ethanol……………………………13
Fig. S15. Absorption spectra of compounds **2-4** in DMF……………………………14
Fig. S16. Absorption spectra of compounds **2-4** in DMSO……………………………14
Fig. S17. Absorption spectra of compounds **2-4** in acetonitrile……………………………15
Table S4. UV-Vis absorption maxima (λ_{Abs}) and logarithms of molar absorption coefficients (logε) of compounds **2-4** in various solvent……………………………15

NMR data of

2

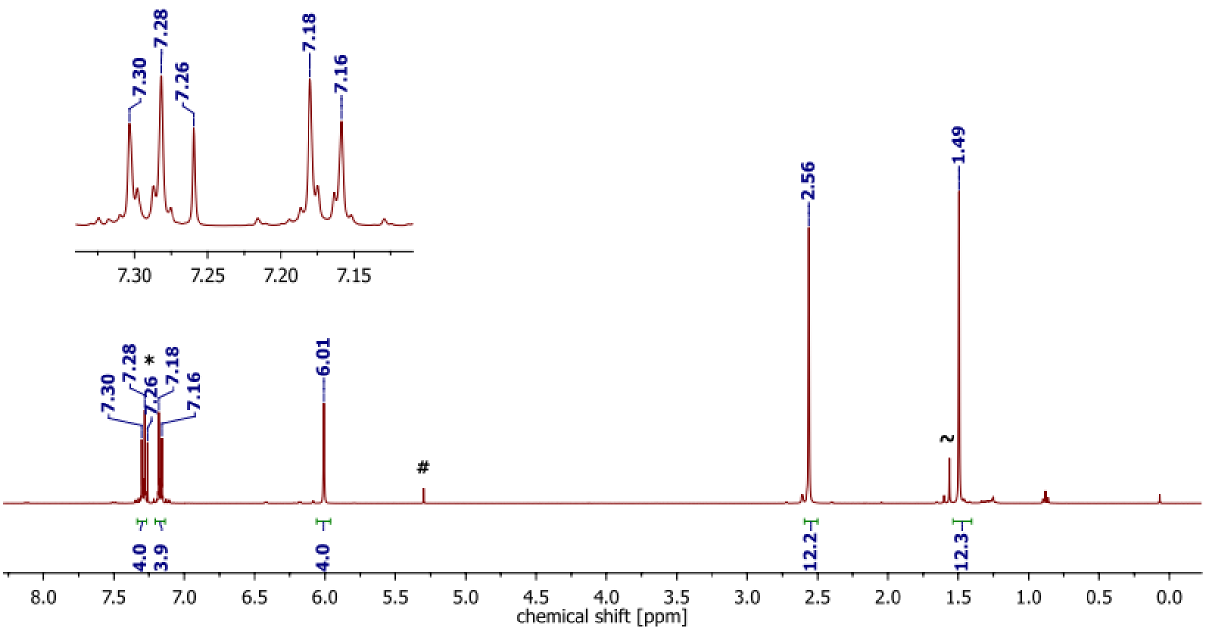

Fig. S1. ^{1}H NMR of **2**. Closely spaced signals are expanded in box above. The symbols *, # and ~ indicate chloroform, dichloromethane and water residual peaks, respectively.

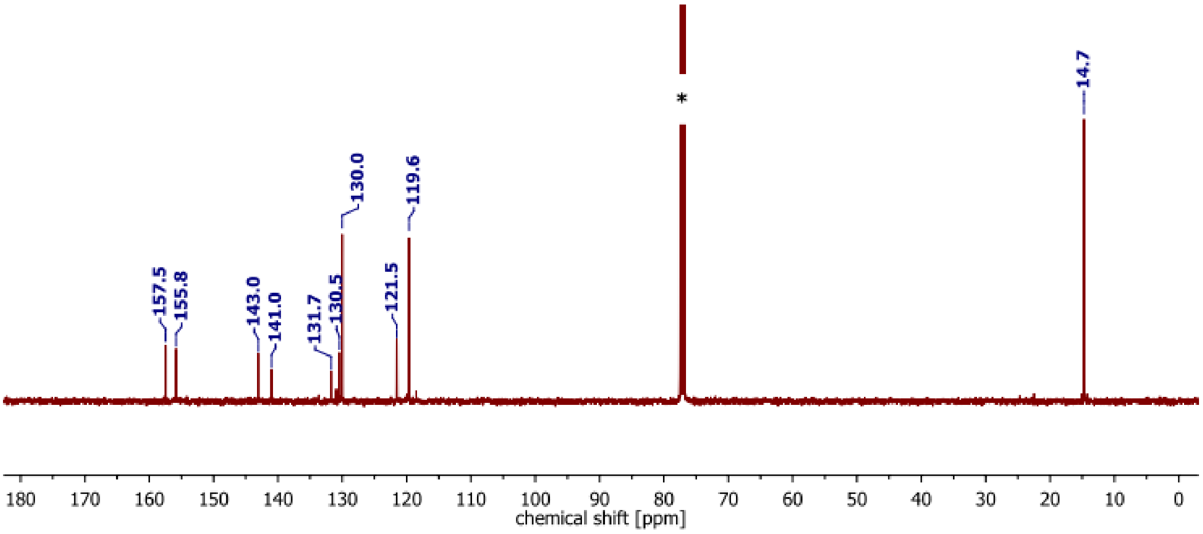

Fig. S2. ^{13}C NMR of **2**. The symbol * indicates chloroform residual peak.

Table S1. NMR data for **2** including key correlations from ^{1}H-^{1}H COSY, ^{1}H-^{13}C HSQC and ^{1}H-^{13}C HMBC spectra.

^{1}H δ_H [ppm]	Multiplicity (J_{H-H} [Hz])	^{1}H-^{1}H COSY δ_H [ppm]	^{1}H-^{13}C HSQC δ_C [ppm]	^{1}H-^{13}C HMBC δ_C [ppm]
1.49	s	2.56, 6.01	14.7	121.5, 131.7, 143.0, 155.8
2.56	s	1.49	14.7	121.5, 143.0, 155.8
6.01	s	1.49	121.5	14.7, 131.7, 143.0, 155.8
7.17	d (9)	7.29	119.6	130.5, 157.5
7.29	d (9)	7.17	130.0	130.5, 141.0, 157.5
δ_C [ppm] from ^{13}C NMR: 157.5, 155.8, 143.0, 141.0, 131.7, 130.5, 130.0, 121.5, 119.6, 14.7				

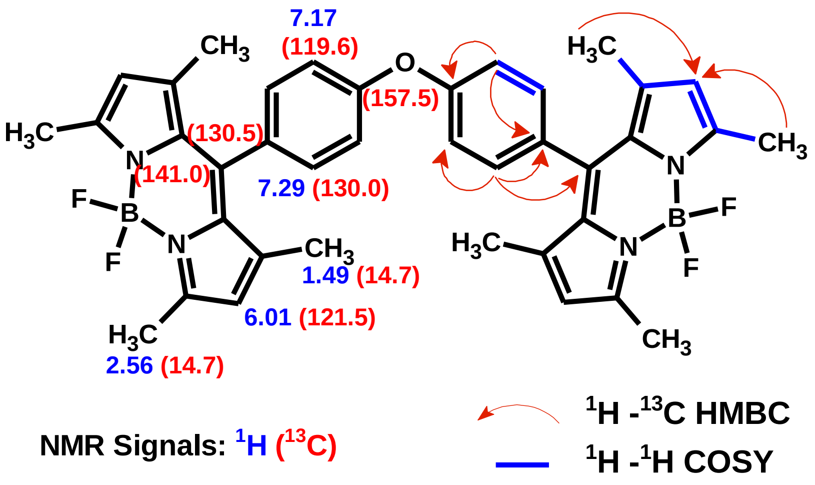

Fig. S3. ^{1}H and (^{13}C) chemical shift values [ppm] and key correlations observed in 2D NMR spectra of **2**.

3

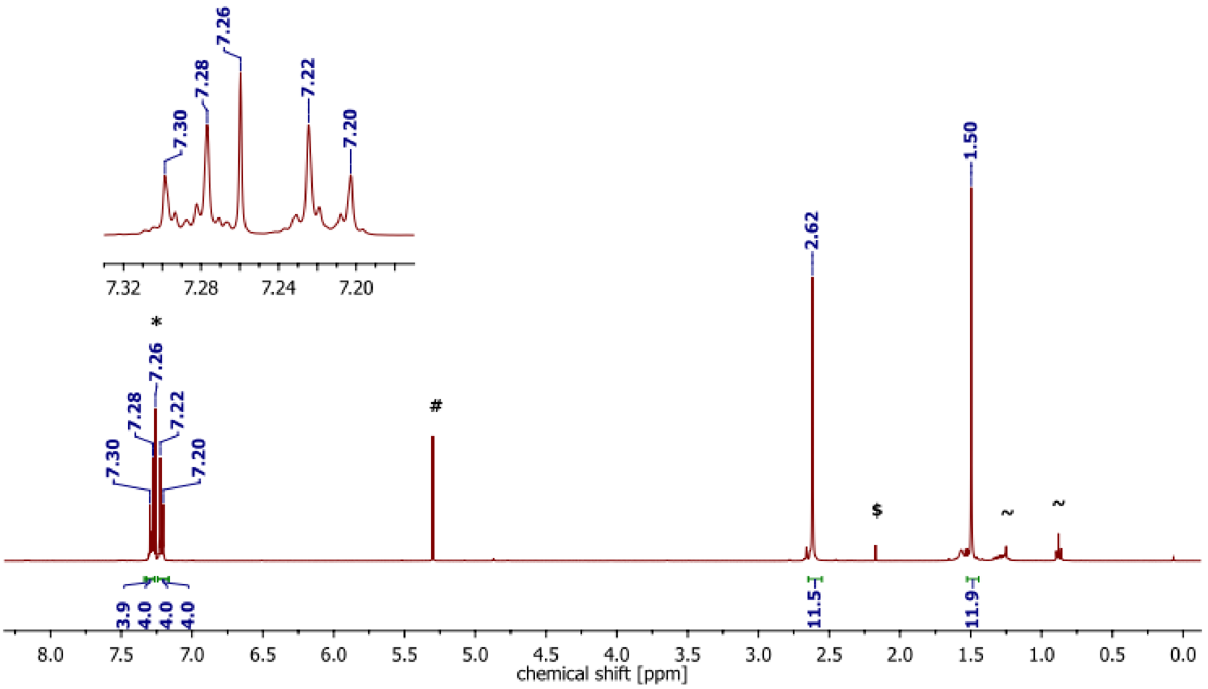

Fig. S4. ^{1}H NMR of **3**. Closely spaced signals are expanded in box above. The symbols *, #, ~ and $ indicate chloroform, dichloromethane, acetone and hexane residual peaks, respectively.

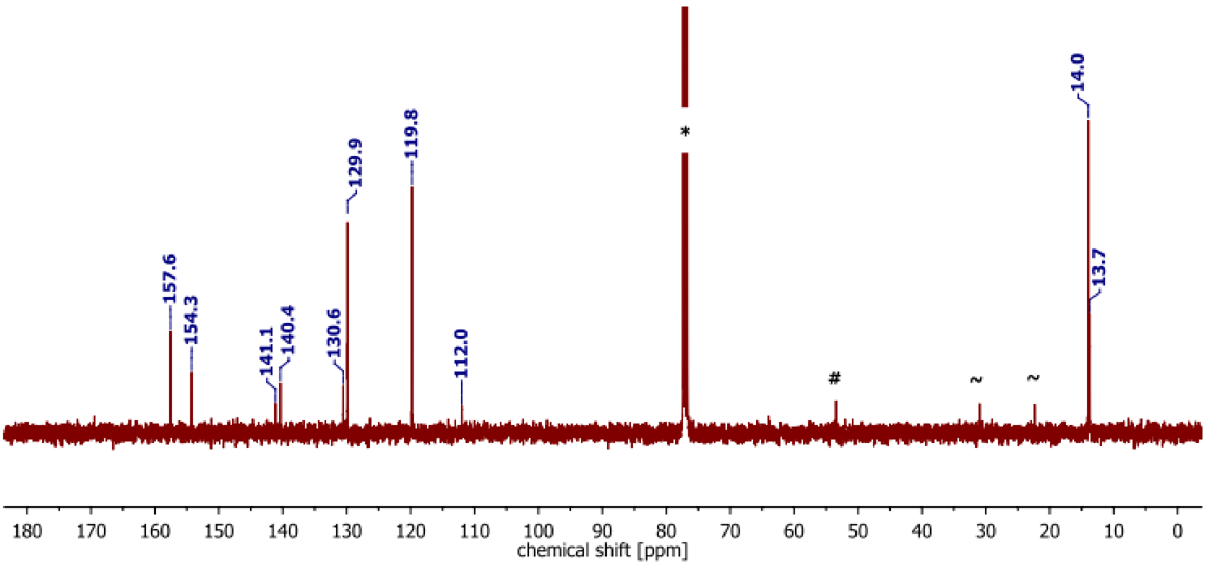

Fig. S5. ^{13}C NMR of **3**. The symbols *, # and ~ indicate chloroform, dichloromethane and hexane residual peaks, respectively.

Table S2. NMR data for **3** including key correlations from ^{1}H-^{1}H COSY, ^{1}H-^{13}C HSQC and ^{1}H-^{13}C HMBC spectra.

^{1}H δ_H [ppm]	Multiplicity ($J_{H\text{-}H}$ [Hz])	^{1}H-^{1}H COSY δ_H [ppm]	^{1}H-^{13}C HSQC δ_C [ppm]	^{1}H-^{13}C HMBC δ_C [ppm]
1.50	s	2.62	14.0	112.0, 130.6, 154.3, 140.4
2.62	s	1.50	13.7	112.0, 154.3, 140.4
7.21	d (9)	7.29	119.8	130.6, 157.6
7.29	d (9)	7.21	129.9	119.8, 141.1, 157.6
δ_C [ppm] from ^{13}C NMR: 157.6, 154.3, 141.1, 140.4. 130.6, 129.9, 119.8, 112.0, 14.0, 13.7				

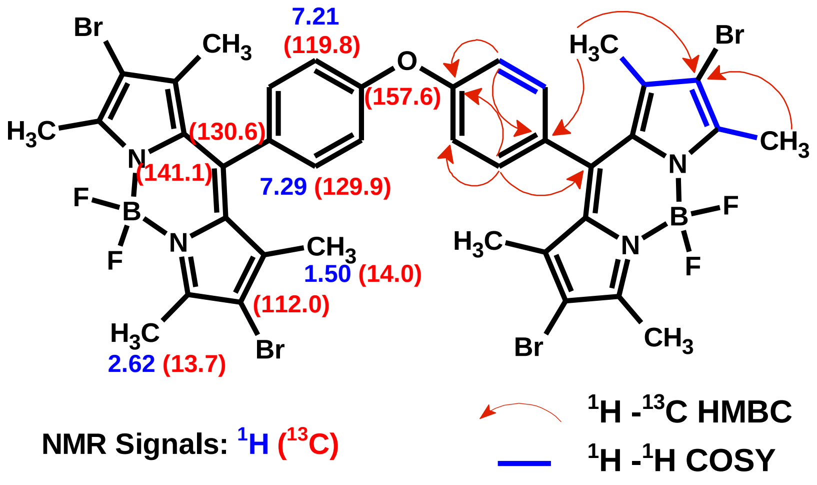

Fig. S6. ^{1}H and (^{13}C) chemical shift values [ppm] and key correlations observed in 2D NMR spectra of **3**.

4

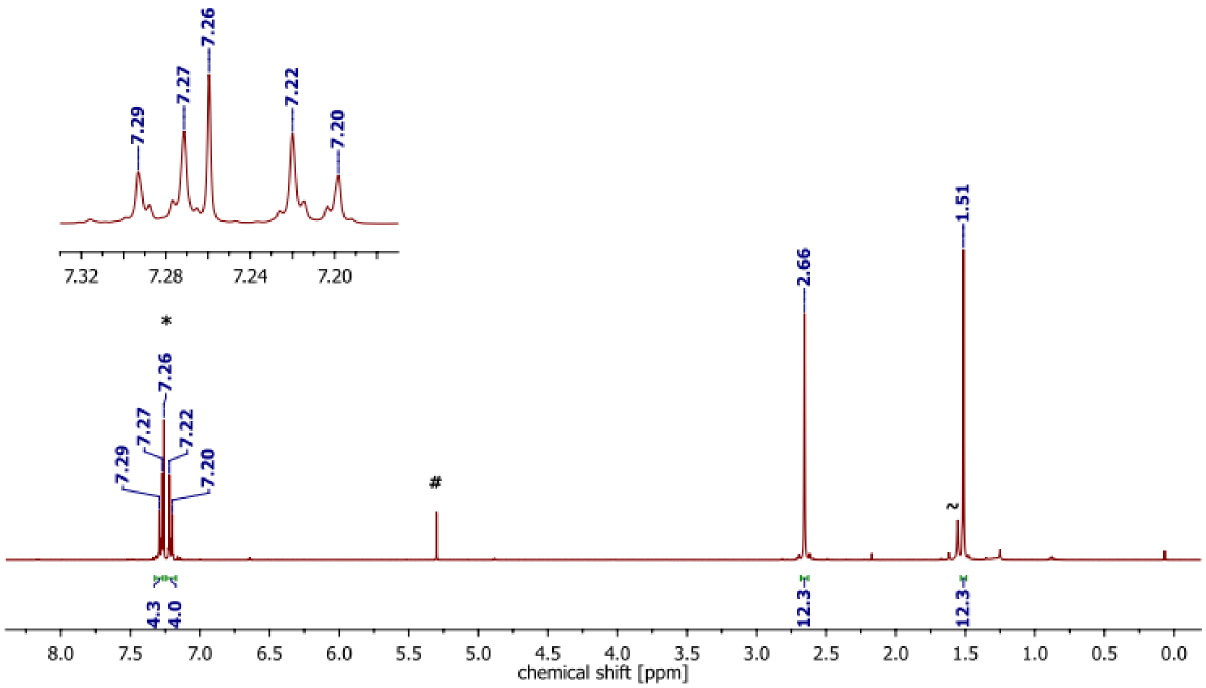

Fig. S7. ^{1}H NMR of **4**. Closely spaced signals are expanded in box above. The symbols *, # and ~ indicate chloroform, dichloromethane and water residual peaks, respectively.

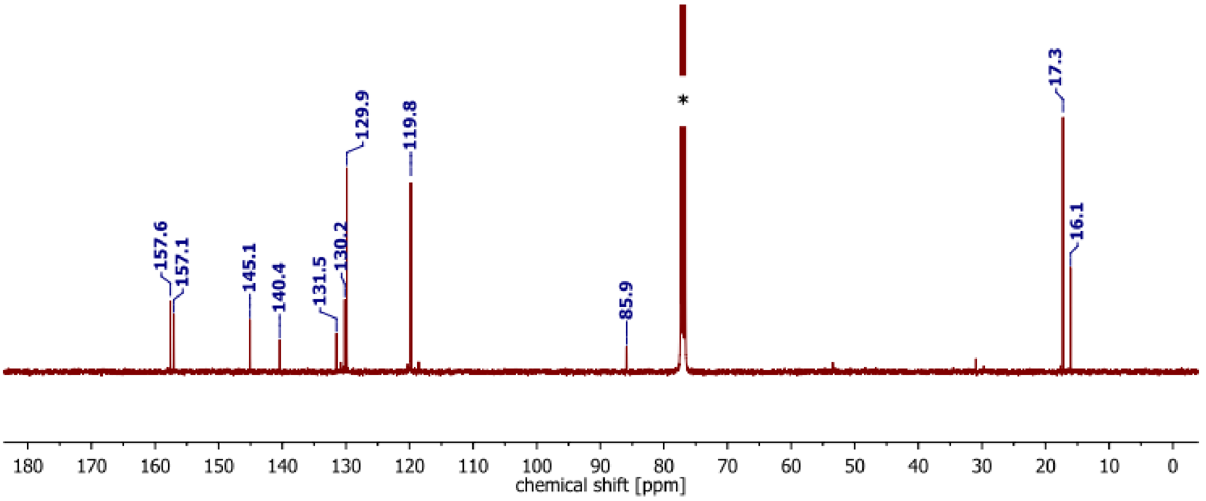

Fig. S8. ^{13}C NMR of **4**. The symbols * indicates chloroform residual peak.

Table S3. NMR data for **4** including key correlations from ^{1}H-^{1}H COSY, ^{1}H-^{13}C HSQC and ^{1}H-^{13}C HMBC spectra.

^{1}H δ_H [ppm]	Multiplicity (J_{H-H} [Hz])	^{1}H-^{1}H COSY δ_H [ppm]	^{1}H-^{13}C HSQC δ_C [ppm]	^{1}H-^{13}C HMBC δ_C [ppm]
1.51	s	2.66	17.3	85.9, 131.5, 145.1, 157.1
2.66	s	1.51	16.1	85.9, 140.4, 145.1, 157.1
7.21	d (9)	7.28	119.8	130.2, 157.6
7.28	d (9)	7.21	129.9	140.4, 157.6
δ_C [ppm] from ^{13}C NMR: 157.6, 157.1, 145.1, 140.4,131.5, 130.2, 129.9, 119.8, 85.9, 17.3, 16.1				

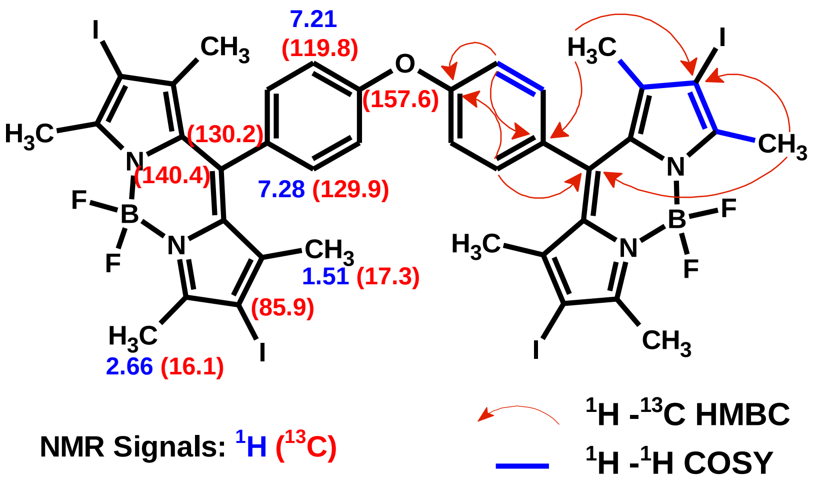

Fig. S9. ^{1}H and (^{13}C) chemical shift values [ppm] and key correlations observed in 2D NMR spectra of **4**.

Mass Spectrometry data of

2

Acquisition Parameter

Source Type	ESI	Ion Polarity	Negative	Set Nebulizer	0.3 Bar
Focus	Active	Set Capillary	3900 V	Set Dry Heater	200 °C
Scan Begin	500 m/z	Set End Plate Offset	-500 V	Set Dry Gas	4.0 l/min
Scan End	1500 m/z	Set Charging Voltage	2000 V	Set Divert Valve	Source
		Set Corona	0 nA	Set APCI Heater	0 °C

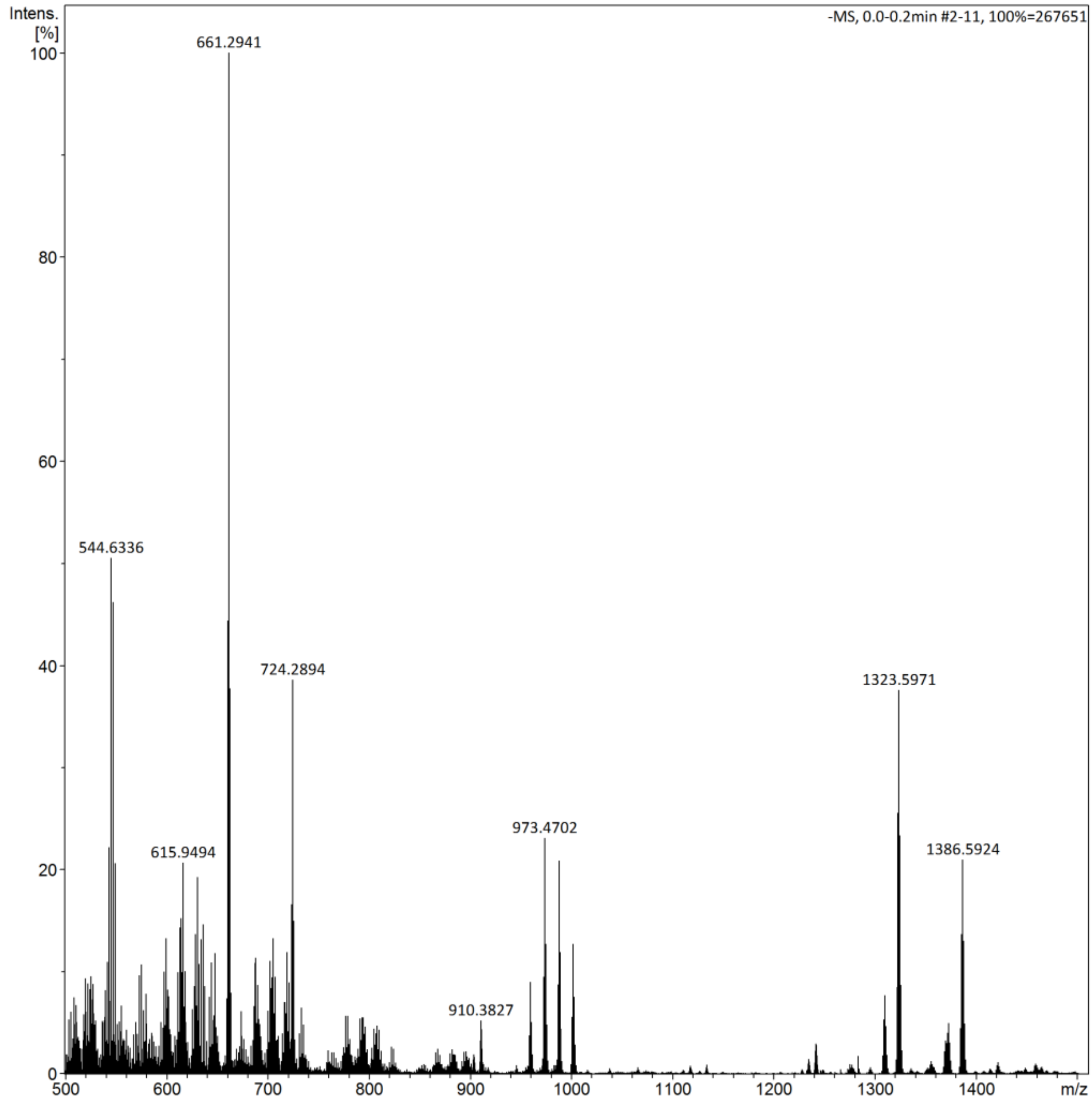

Fig. S10. HRMS spectrum of **2**.

3

Acquisition Parameter

Source Type	ESI	Ion Polarity	Negative	Set Nebulizer	0.3 Bar
Focus	Active	Set Capillary	3900 V	Set Dry Heater	200 °C
Scan Begin	500 m/z	Set End Plate Offset	-500 V	Set Dry Gas	4.0 l/min
Scan End	1500 m/z	Set Charging Voltage	2000 V	Set Divert Valve	Source
		Set Corona	0 nA	Set APCI Heater	0 °C

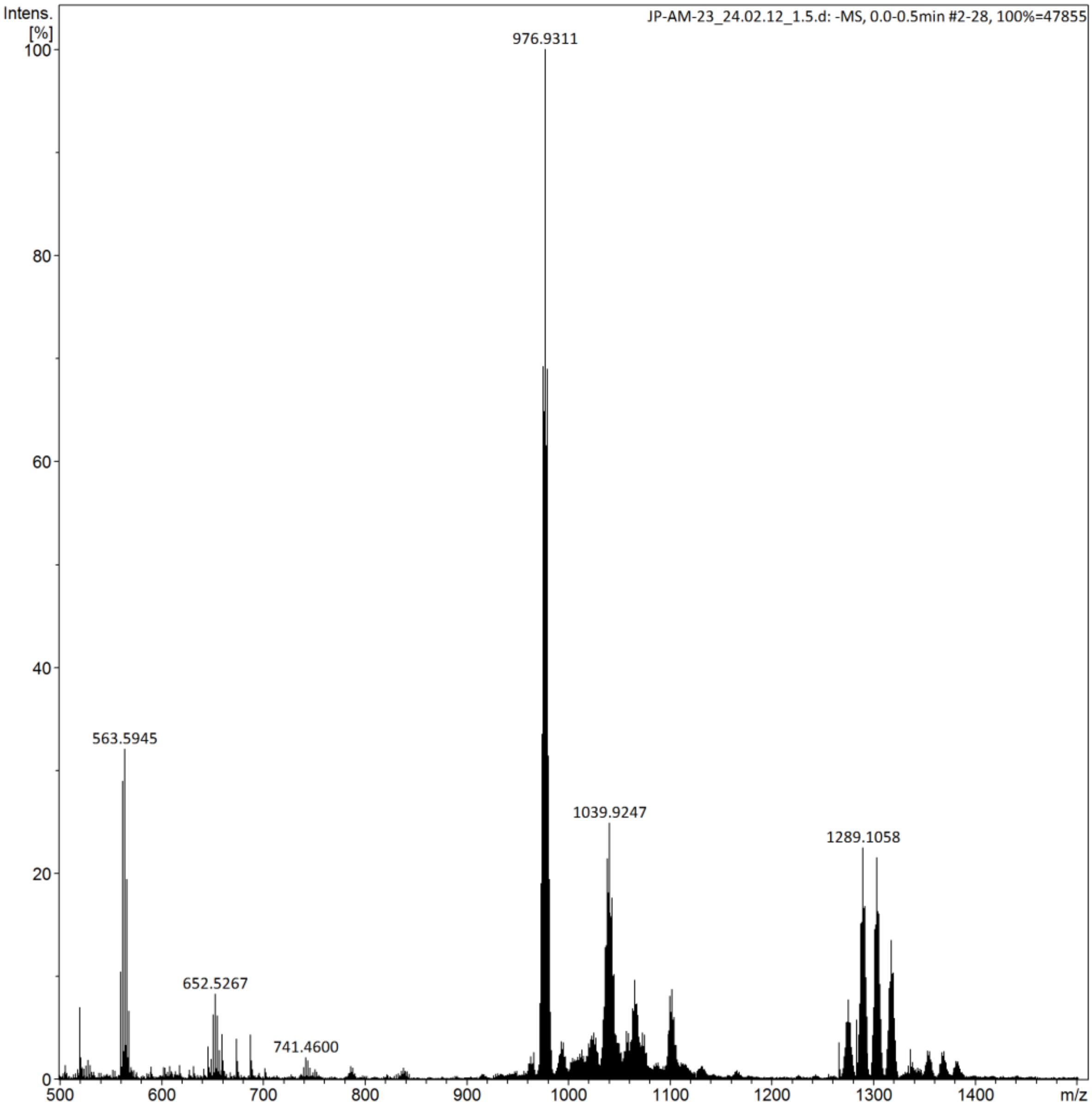

Acquisition Parameter

Source Type	ESI	Ion Polarity	Negative	Set Nebulizer	0.3 Bar
Focus	Active	Set Capillary	3900 V	Set Dry Heater	200 °C
Scan Begin	500 m/z	Set End Plate Offset	-500 V	Set Dry Gas	4.0 l/min
Scan End	1500 m/z	Set Charging Voltage	2000 V	Set Divert Valve	Source
		Set Corona	0 nA	Set APCI Heater	0 °C

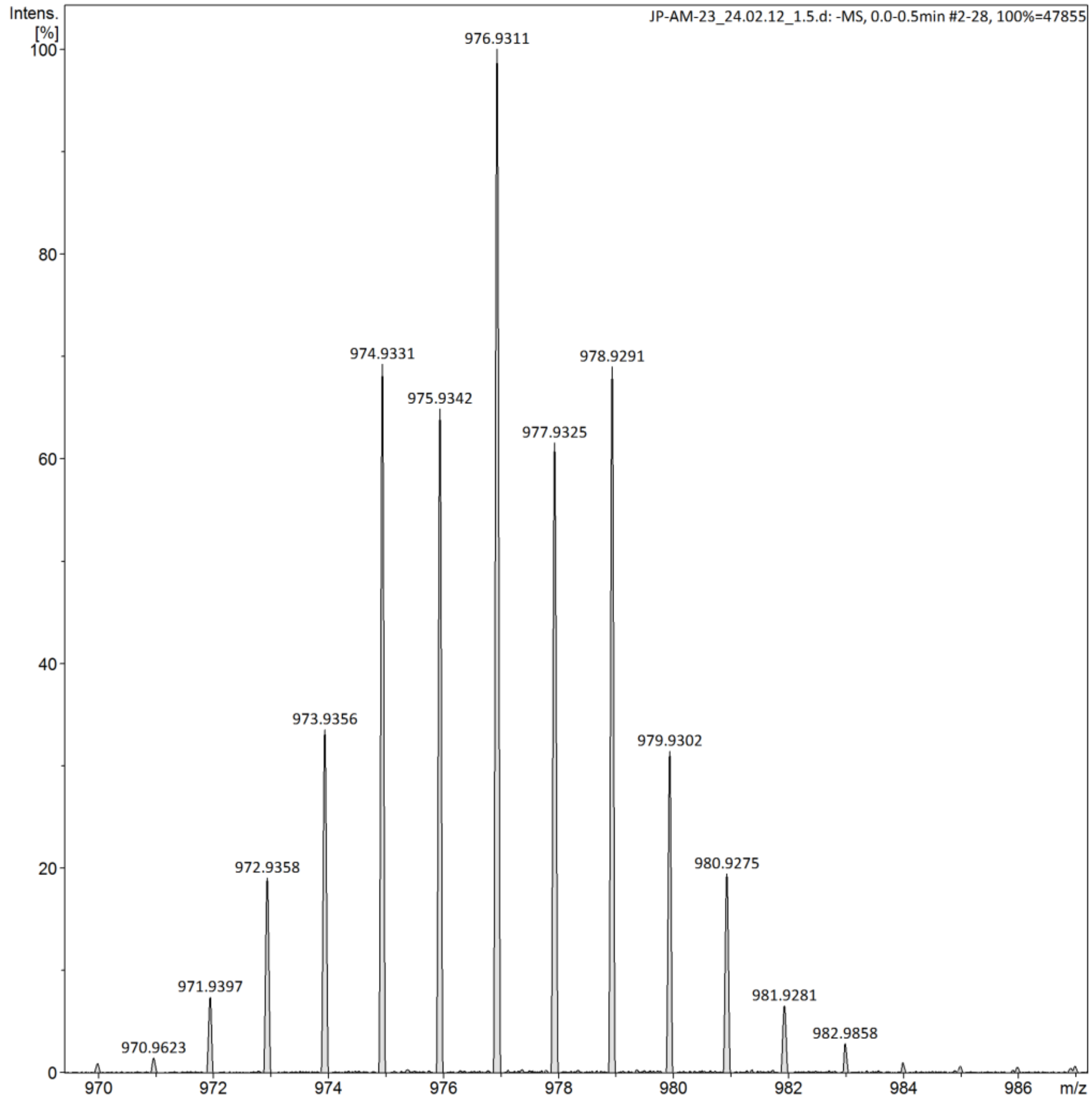

Fig. S11. HRMS spectra of **3**.

4

Acquisition Parameter

Source Type	ESI	Ion Polarity	Negative	Set Nebulizer	0.3 Bar
Focus	Active	Set Capillary	3800 V	Set Dry Heater	200 °C
Scan Begin	350 m/z	Set End Plate Offset	-500 V	Set Dry Gas	4.0 l/min
Scan End	1700 m/z	Set Charging Voltage	2000 V	Set Divert Valve	Source
		Set Corona	0 nA	Set APCI Heater	0 °C

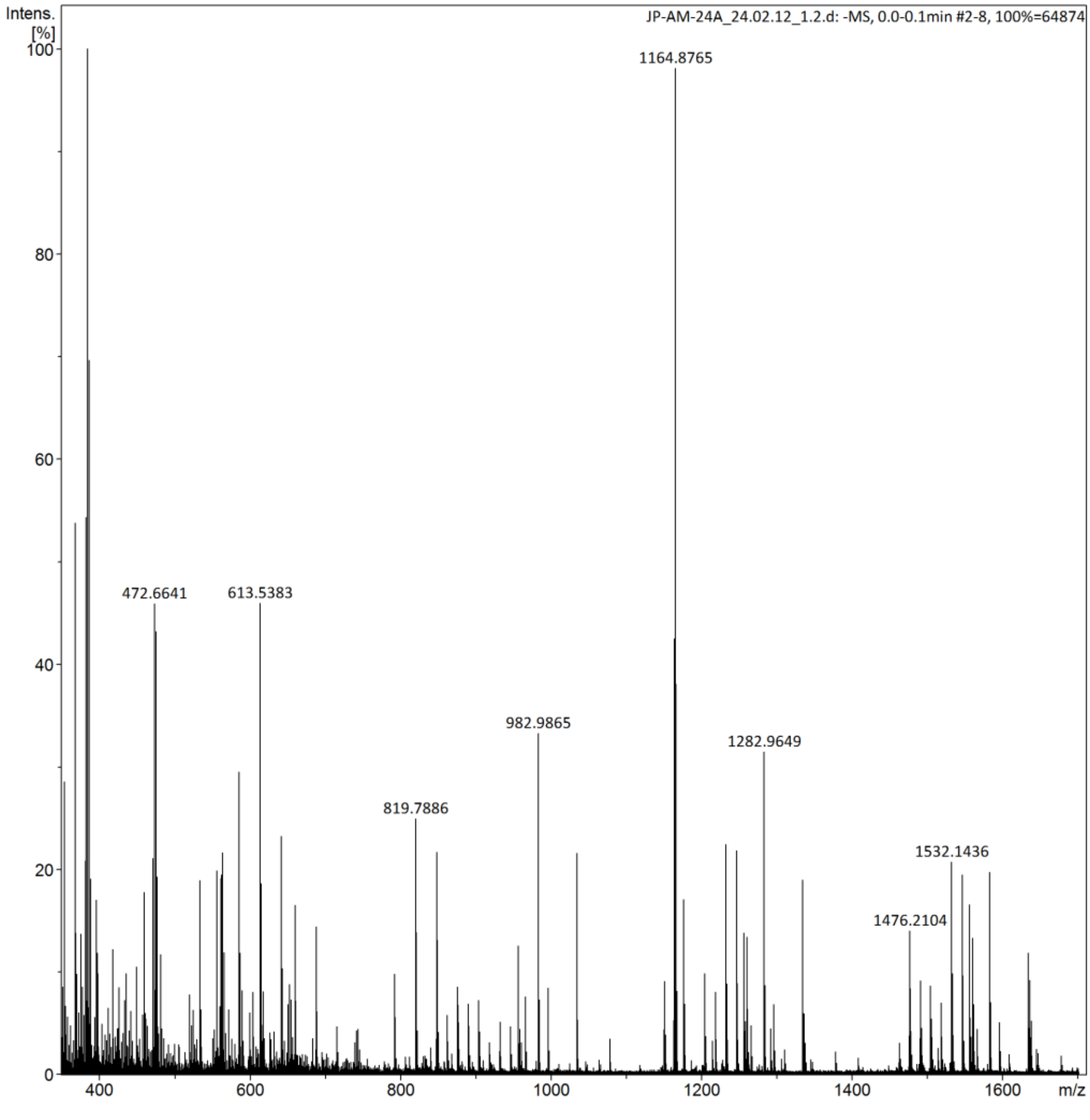

Fig. S12. HRMS spectrum of **4**.

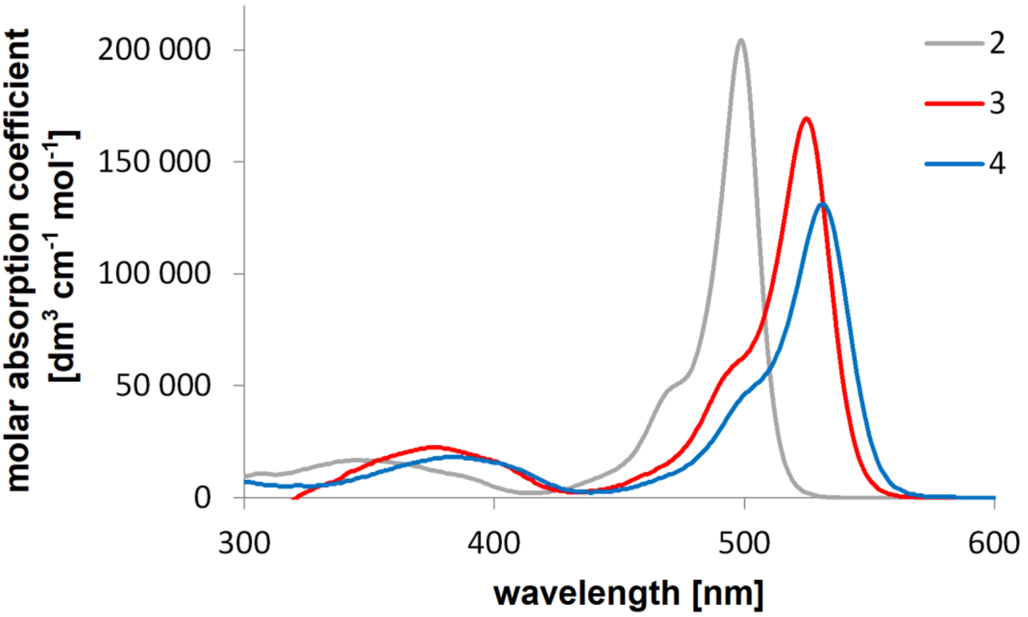

Fig S13. Absorption spectra of compounds **2-4** in methanol.

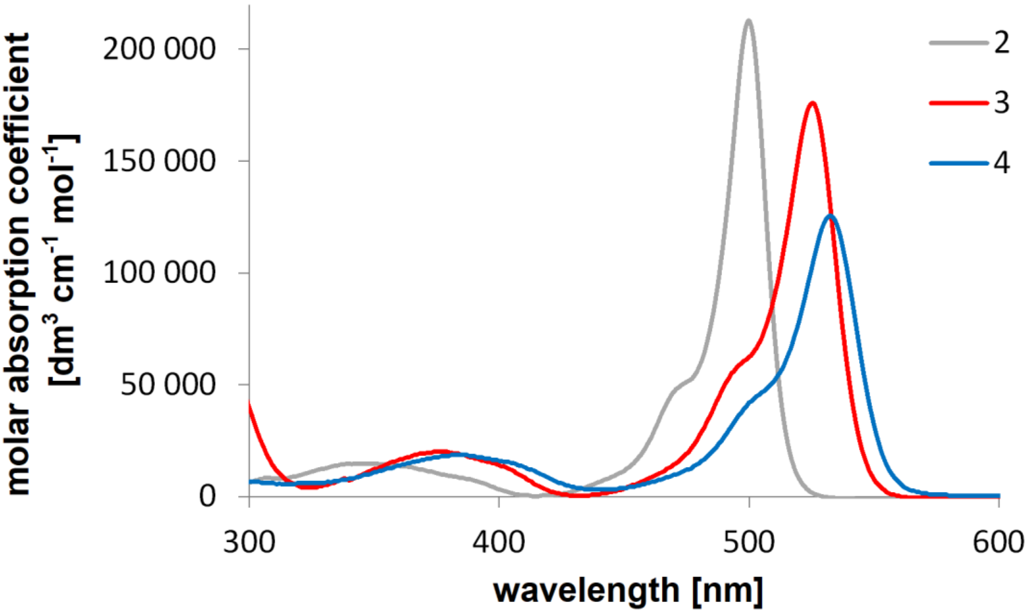

Fig S14. Absorption spectra of compounds **2-4** in ethanol.

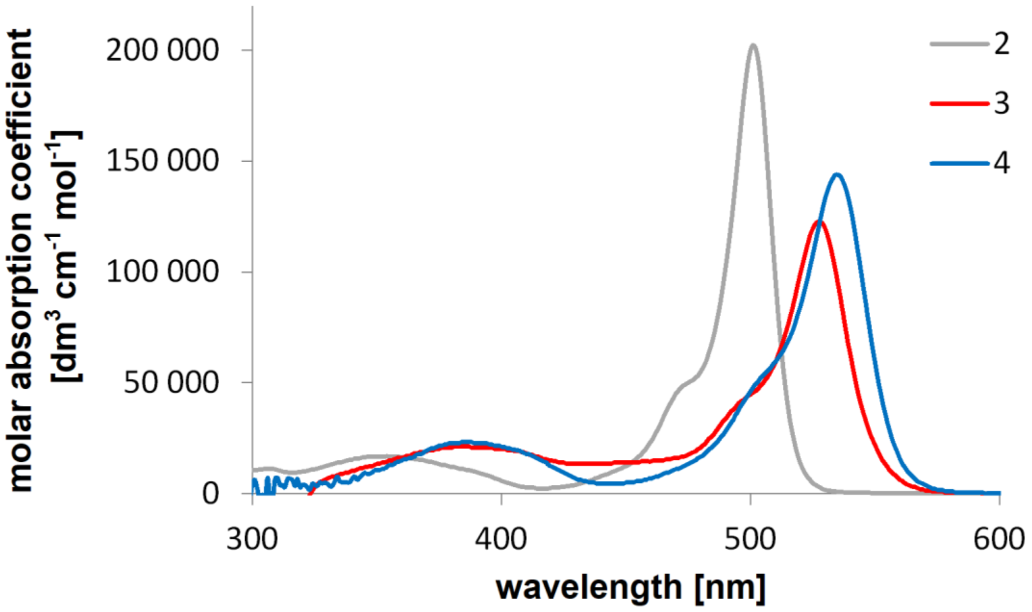

Fig S15. Absorption spectra of compounds **2-4** in DMF.

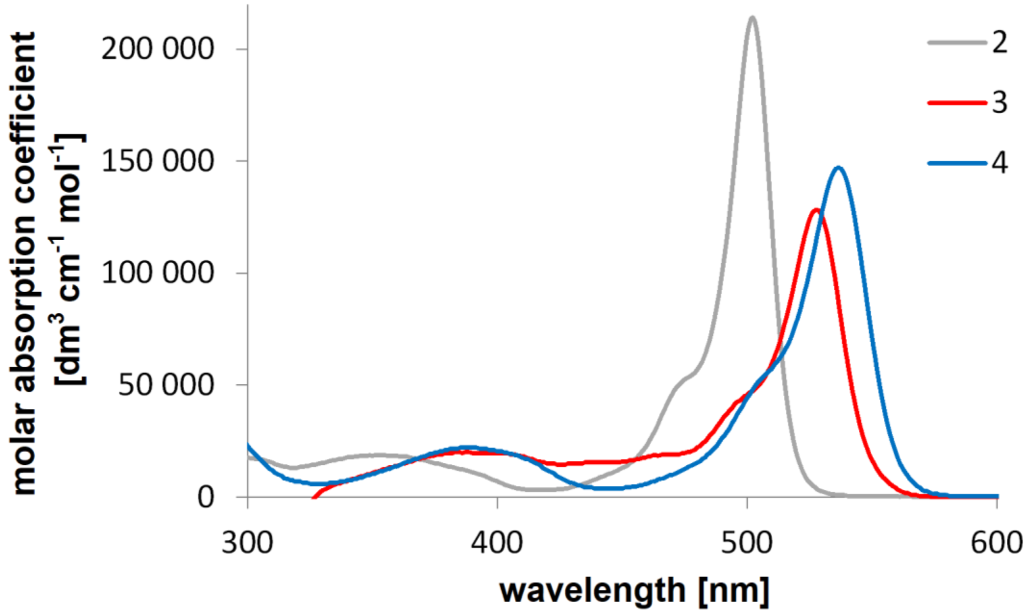

Fig S16. Absorption spectra of compounds **2-4** in DMSO.

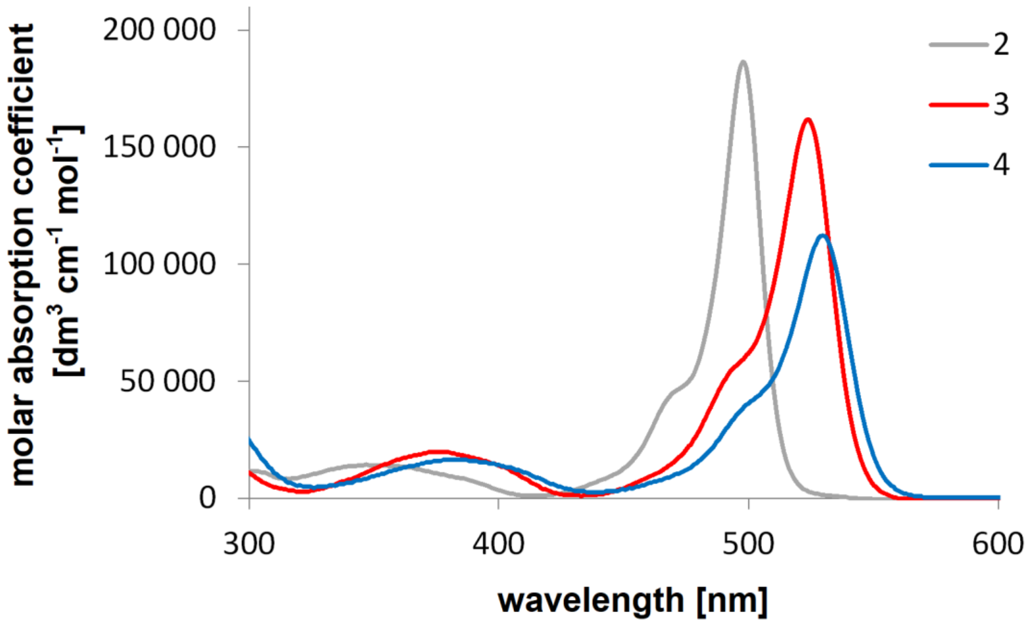

Fig S17. Absorption spectra of compounds **2-4** in acetonitrile.

Table S4. UV-Vis absorption maxima (λ_{Abs}) and logarithms of molar absorption coefficients (log ε) of compounds **2-4** in various solvents

		BODIPY					
BODIPY	solvent	**CH_2Cl_2**	**CH_3OH**	**C_2H_5OH**	**DMF**	**DMSO**	**CH_3CN**
**2**	λ_1 (log ε)<br>λ_2 (log ε)	347 (4.31)<br>502 (5.32)	345 (4.23)<br>499 (5.31)	346 (4.17)<br>500 (5.33)	354 (4.23)<br>501 (5.31)	353 (4.28)<br>502 (5.33)	347 (4.16)<br>498 (5.27)
**3**	λ_1 (log ε)<br>λ_2 (log ε)	378 (4.32)<br>529 (5.24)	376 (4.35)<br>525 (5.23)	377 (4.31)<br>526 (5.25)	384 (4.33)<br>527 (5.09)	387 (4.31)<br>528 (5.11)	376 (4.30)<br>524 (5.21)
**4**	λ_1 (log ε)<br>λ_2 (log ε)	386 (4.21)<br>535 (5.20)	383 (4.26)<br>531 (5.12)	384 (4.28)<br>532 (5.10)	386 (4.37)<br>535 (5.16)	388 (4.35)<br>536 (5.17)	382 (4.22)<br>530 (5.05)
